# Universal Access to Protease Chemiluminescent Probes through Solid-Phase Synthesis

**DOI:** 10.1021/acs.bioconjchem.1c00384

**Published:** 2021-09-22

**Authors:** Maria Ponomariov, Doron Shabat, Ori Green

**Affiliations:** † School of Chemistry, Raymond and Beverly Sackler Faculty of Exact Sciences, Tel-Aviv University, Tel Aviv 69978, Israel

## Abstract

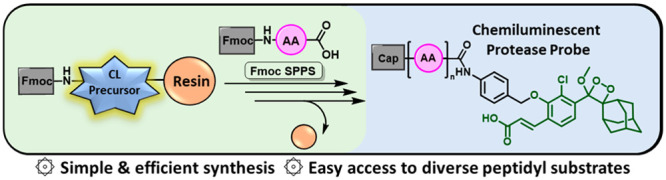

Protease chemiluminescent probes exhibit extremely high detection sensitivity for monitoring activity of various proteolytic enzymes. However, their synthesis, performed in solution, involves multiple synthetic and purification steps, thereby generating a major limitation for rapid preparation of such probes with diverse substrate scope. To overcome this limitation, we developed a general solid-phase-synthetic approach to prepare chemiluminescent protease probes, by peptide elongation, performed on an immobilized chemiluminescent enol-ether precursor. The enol-ether precursor is immobilized on a 2-chlorotrityl-chloride resin through an acrylic acid substituent by an acid-labile ester linkage. Next, a stepwise elongation of the peptide is performed using standard Fmoc solid-phase peptide synthesis. After cleavage of the peptide-enol-ether precursor from the resin, by hexafluoro-iso-propanol, a simple oxidation of the enol-ether yields the final chemiluminescent dioxetane protease probe. To validate the applicability of the methodology, two chemiluminescent probes were efficiently prepared by solid-phase synthesis with dipeptidyl substrates designed for activation by aminopeptidase and cathepsin-B proteases. A more complex example was demonstrated by the synthesis of a chemiluminescent probe for detection of PSA, which includes a peptidyl substrate of six amino acids. We anticipate that the described methodology would be useful for rapid preparation of chemiluminescent protease probes with vast and diverse peptidyl substrates.

Proteases are a class of enzymes that are involved in almost every biological signaling and regulation processes in living systems.^1,2^ The ability of these enzymes, to cleave peptide bonds, is crucial for protein turnover in cells.^3^ In addition, proteases are involved not only in protein degradation but also in protein activation. Therefore, proteases are strongly associated with growth, cell division, differentiation, migration, and signaling. The classification of proteases is usually determined by their proteolytic mechanism, which involves various amino acid residues and includes cysteine-proteases, serine-proteases, threonine-proteases, and metalloproteases. Although these proteases mechanistically differ from one another, they all share one key principle: the ability to hydrolyze a specific amide bond in their peptidyl substrate. This amide bond breakdown is the central feature that enables scientists to design chemical tools for selective monitoring of protease activity.^4^

The most common method for monitoring protease activity is based on optical substrates, where fluorescence is the prominent modality.^5,6^ The general design and synthesis of turn-on fluorescent probes are presented in Figure 1A. Upon proteolytic cleavage of an amide bond located between a specific peptide and a fluorescent dye, an increase of a fluorescent signal, correlating to the catalytic activity of the protease, is produced. Such probes have been widely used to determine substrate specificity of proteases. In addition, they also provided valuable insights in regard to biological functions of proteases and thus led to the discovery of new inhibitors and drugs.

**Figure 1:**
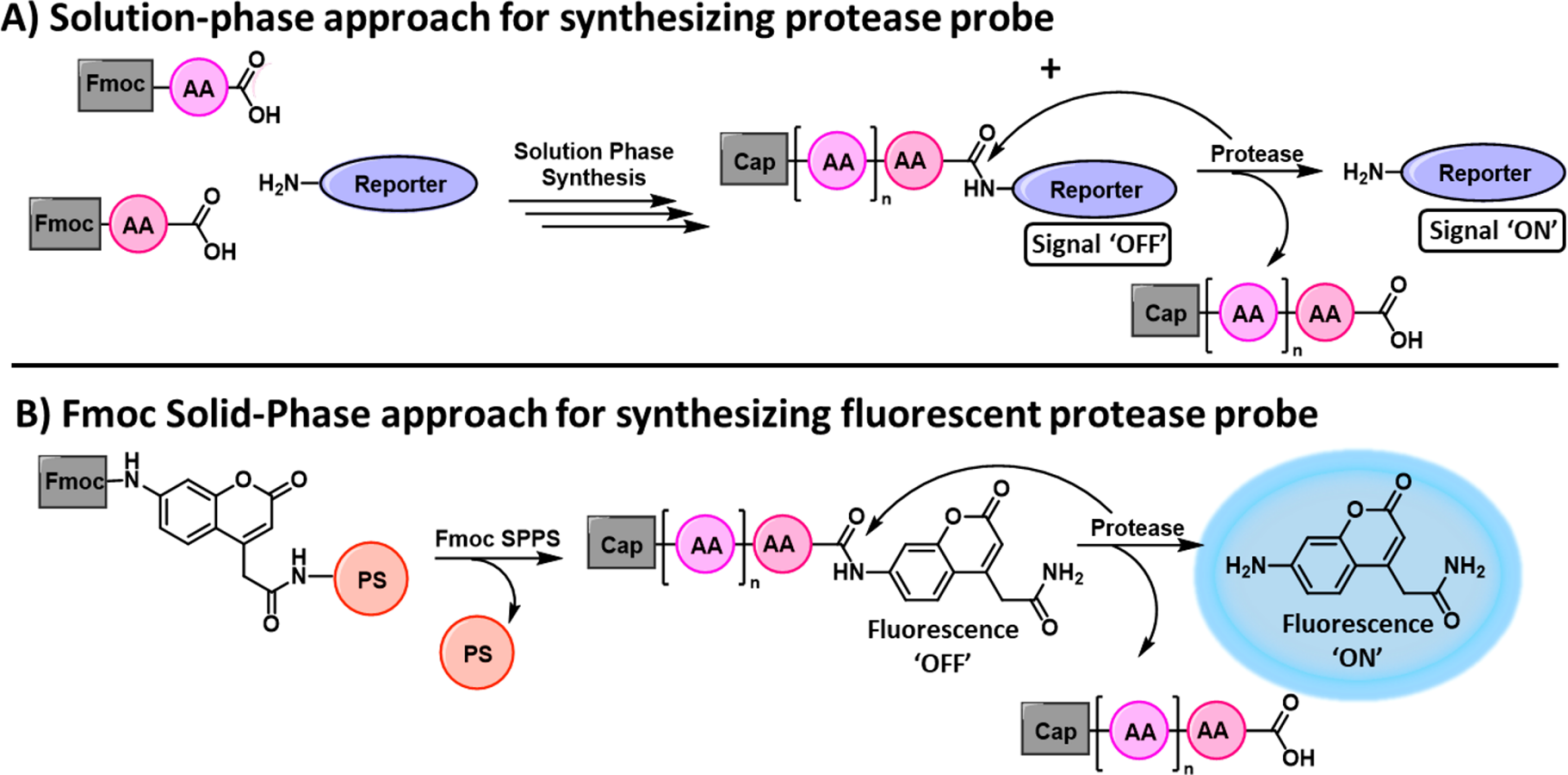
(A) Solution-phase synthesis approach to fluorescent protease probes. (B) Solid-phase synthesis approach to fluorescent protease probes.

The synthesis of fluorogenic protease probes is usually performed in solution, where the C-terminus of a premade peptidyl substrate is coupled with an amino group of the fluorogenic dye (Figure 1A).^7,8^ This synthetic strategy consumes much time and resources, and requires challenging purification steps, especially when the peptidyl substrate involves the use of protecting groups.^9^ In addition, the synthesis in solution cannot be automated or be applied to efficiently prepare combinatorial substrate libraries for fluorescent probes.^3,10^ To overcome this limitation, researchers have developed a solid-phase peptide synthesis (SPPS) on a resin with an anchored fluorescent dye. The SPPS, introduced by Merrifield in the 1960s, is a fast and robust technique to synthesize peptides.^11,12^ Unlike synthesis in solution, SPPS can be automated and therefore can be used to create vast libraries of peptidyl substrates, in a method known as Positional Scanning Synthetic Combinatorial Libraries (PS-SCLs).^13,14^

In 2002, the Ellman group reported the synthesis of a Rink Amide AM resin, immobilized with 7-amino-4-carbamoylmethylcoumarin fluorescent dye.^15^ The amino functional group of the dye was used as a handle to perform SPPS. The protease fluorescent probe was directly obtained after cleavage of the peptide–dye conjugate from the resin (Figure 1B). Such a simple and elegant synthetic strategy was found to be extremely efficient and enabled the straightforward preparation of PS-SCLs. Over the years, this methodology has proven to be particularly useful, contributing to the discovery of numerous selective substrates and inhibitors, and thereby helping to elucidate many key biological functions of proteases.^16−19^

The most common fluorogenic reporter used in SPPS for preparation of protease probes is the 7-amino-4-methyl-coumarin dye (AMC). However, while fluorescence has some important benefits, this modality also has limitations, since the need for an external light source for excitation hampers the sensitivity of the assay. In addition, the use of AMC, which emits light in the blue region, is not optimal for imaging applications due to the low tissue-permeability of short wavelengths. Recently, our group developed chemiluminescent luminophores that are highly emissive under physiological conditions.^20−22^ Molecular probes composed of these luminophores were found to be suitable for monitoring enzymatic activity with great sensitivity, both *in vitro* and *in vivo*.^23−25^ The general design and activation pathway for such chemiluminescent probes is illustrated in Figure 2. Remarkably, protease probes based on our chemiluminescent dioxetane luminophore exhibited up to 16,000-fold higher sensitivity in comparison to analogous fluorescent probes based on AMC dye.^26−31^

**Figure 2:**
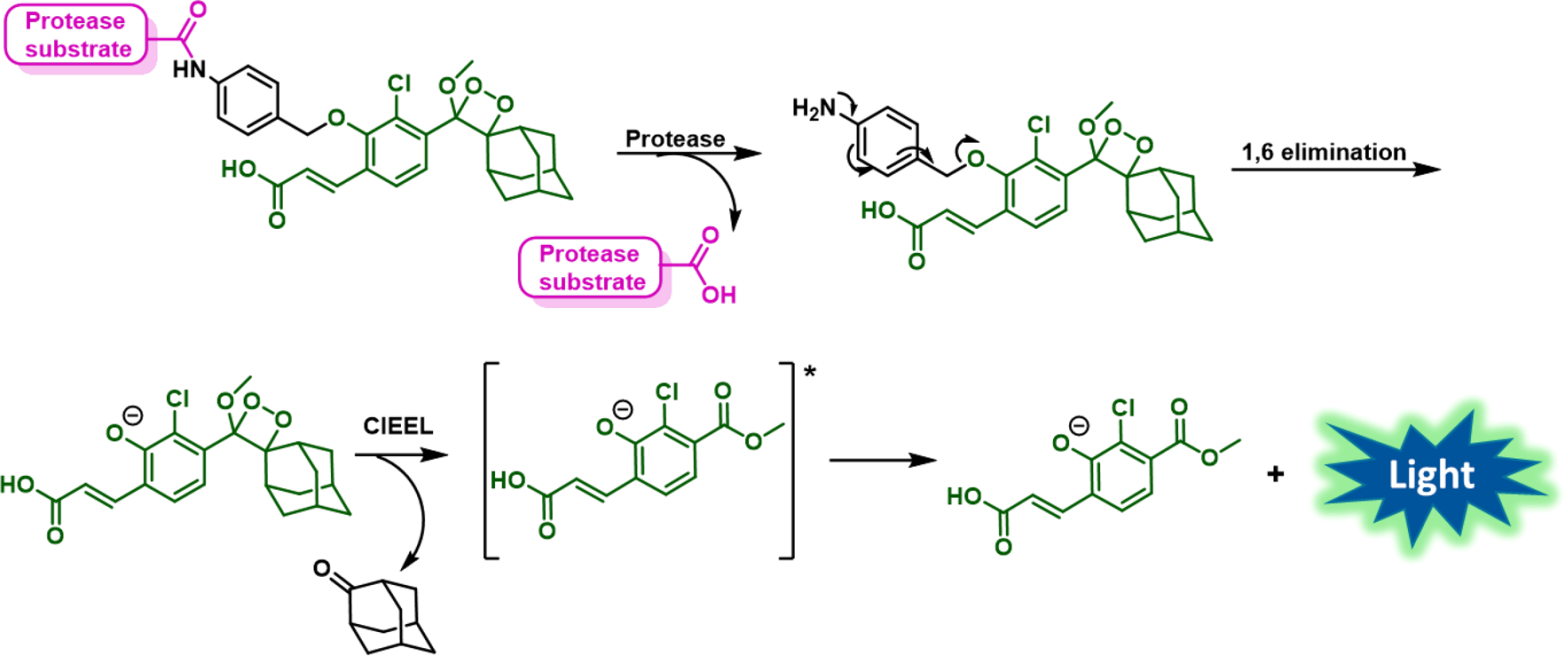
General activation and chemiexcitation pathway of protease chemiluminescent probes.

Although chemiluminescent probes for proteases showed unprecedented detection sensitivity, their synthesis, which is performed in solution and involves multiple purification steps, poses a major limitation for obtaining a diverse substrate scope. To overcome this limitation, we report here a general solid-phase synthesis approach to prepare chemiluminescent protease probes by peptide elongation on an immobilized chemiluminescent precursor. The establishment of such a platform could contribute immensely to obtaining positional libraries for protease chemiluminescent probes, suitable for a wide range of applications.

The general layout of our approach is described in Figure 3. Our methodology relies on the initial preparation of a phenol enol-ether (dioxetane precursor) building block, attached through a *p*-aminobenzyl alcohol (PABA) linker to the C-terminus of the first Fmoc-protected amino acid (Fmoc-AA) of the desired sequence. This building block is then immobilized on a 2-chlorotrityl-chloride resin through the acrylic acid substituent by an acid-labile ester linkage. Next, a stepwise elongation of the peptide is performed using standard Fmoc SPPS. After cleavage of the peptide-enol-ether from the resin, a simple oxidation step is performed to generate the final dioxetane protease probe. The immobilization of the enol-ether precursor on 2-chlorotrityl-chloride resin allowed us to perform the cleavage step by using the mild acid, hexafluoro-iso-propanol (HFIP). Such a mild acid can selectively cleave the trityl-ester linkage, while keeping the relative unstable enol-ether functionality intact.

**Figure 3:**
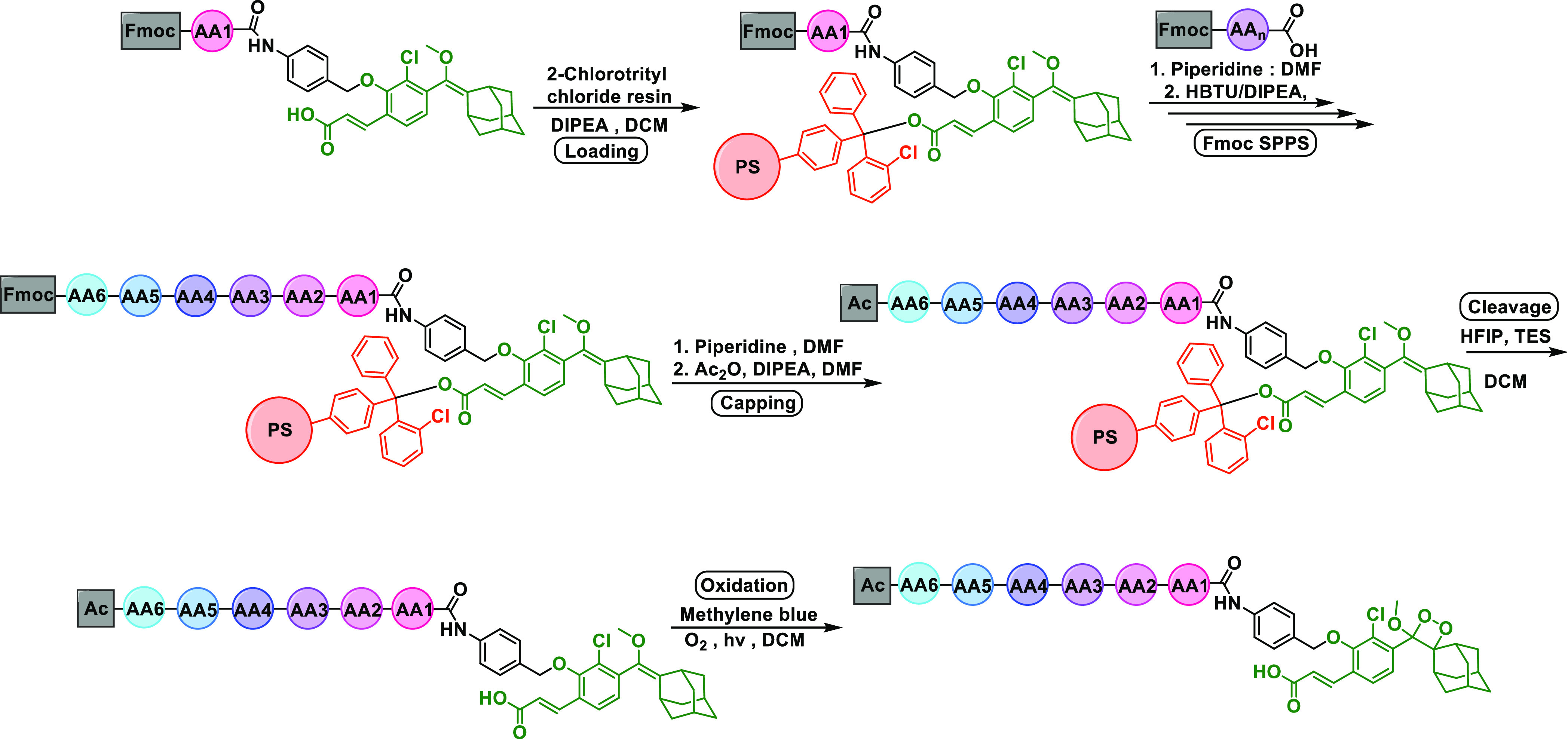
Schematic solid-phase synthesis of protease probes using an anchored chemiluminescent precursor (enol-ether) on 2-chlorotrityl-chloride resin.

The synthetic pathway for the preparation of an enol-ether building block, attached to one Fmoc-AA, is described in the Supporting Information (see Scheme S1). Following this synthetic route, we synthesized building blocks **1**, **2**, and **3** (Figure 4A), enol-ethers preloaded with alanine (Ala), lysine (Lys), and glutamine (Gln). When Lys was applied, an Alloc protecting group was used to protect the ε-amino side chain (see SI for synthetic route). We chose to focus on the amino acids Ala, Lys, and Gln, since they are present in the peptidyl substrate P1 position of several known proteases.

**Figure 4:**
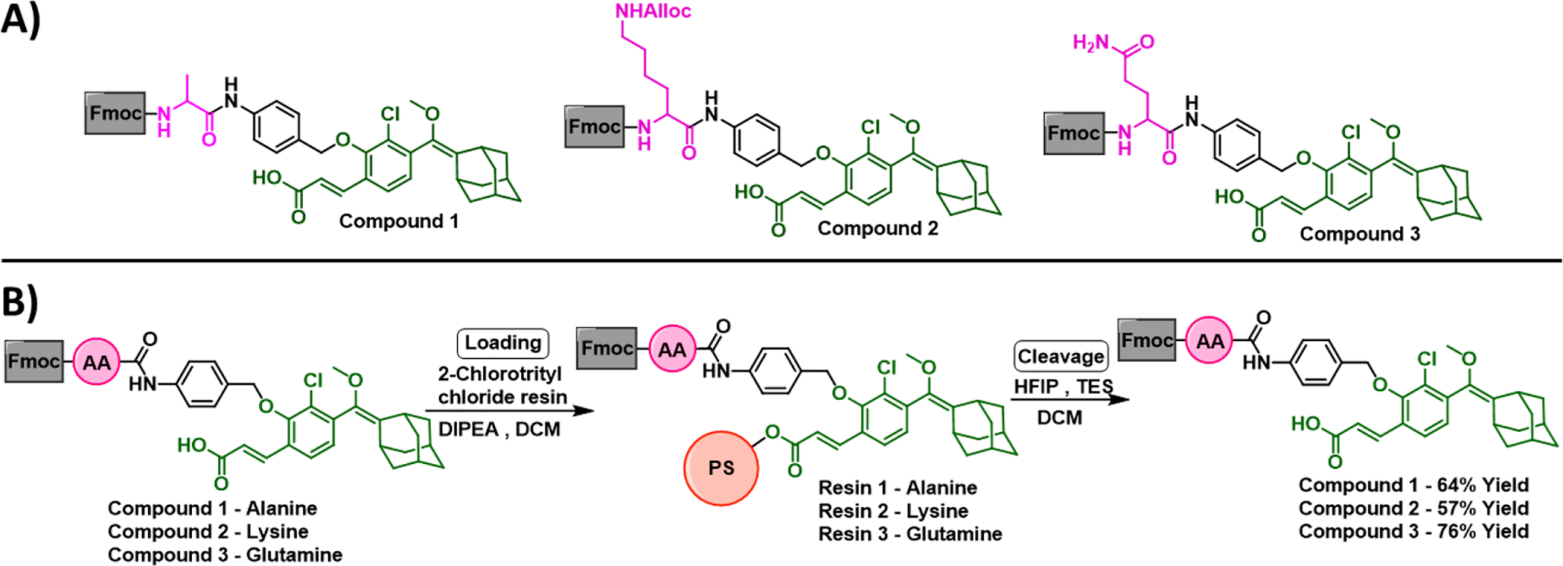
(A) Chemical structures of ready-to-load enol-ethers attached with the Fmoc-amino acids: alanine, lysine, and glutamine. (B) Synthesis of resins **1**, **2**, and **3** and their loading efficiency.

To test our strategy, the loading of building blocks **1**, **2**, and **3** on the 2-chlorotrityl-chloride resin, using *N*,*N*-diisopropylethylamine (DIPEA) as a base and dichloromethane (DCM) as a solvent, was followed by a capping step using a solution of MeOH, DIPEA, and DCM. After several rounds of washing, the resin was treated with the cleavage cocktail. This step was followed by recovery and purification of the starting materials (building blocks **1**, **2**, and **3**). The final yields of this process are depicted in Figure 4B. All three building blocks were loaded and cleaved with decent yields. This initial evaluation provided a clear indication that the enol-ether functionality is compatible with the loading and cleavage conditions.

With the resin-immobilized enol-ethers in hand (Resins **1**, **2**, and **3**), we sought to evaluate our approach, to prepare chemiluminescent protease probes through solid-phase synthesis. For simplicity, we initially chose to prepare protease chemiluminescent probes equipped with simple dipeptidyl triggering substrates. Two such probes were prepared. The first one, with the dipeptidyl substrate Ser-Ala, is a probe for detection of aminopeptidase (AMP) proteolytic activity.^32^ SPPS using Resin **1** afforded **Enol-ether 1** in high purity with a yield of 67% after cleavage. Oxidation of this enol-ether by singlet oxygen afforded the aminopeptidase chemiluminescent probe (**APCP**) in 91% yield (Figure 5A). Overall, **APCP** was synthesized using SPPS with a total yield of 61% and with high purity (SI Figure S10). The second probe that was prepared by this method, with a dipeptidyl substrate, is a probe aimed for activation by the protease cathepsin B (Cat-B). Out of several known peptidyl substrates of Cat-B, we chose to focus on the dipeptidyl substrate Ac-Phe-Lys, which was demonstrated in numerous examples of imaging applications.^8,33−35^ SPPS using Resin **2**, followed by cleavage and removal of the Alloc protecting group using Pd^0^, afforded **Enol-ether 2** in 93% yield. Oxidation of this enol-ether by singlet oxygen gave the Cat-B chemiluminescent probe (**CBCP**) in 59% yield after purification (Figure 5B). Overall, probe **CBCP** was synthesized using SPPS with a total yield of 63% and with high purity (SI Figure S12).

**Figure 5:**
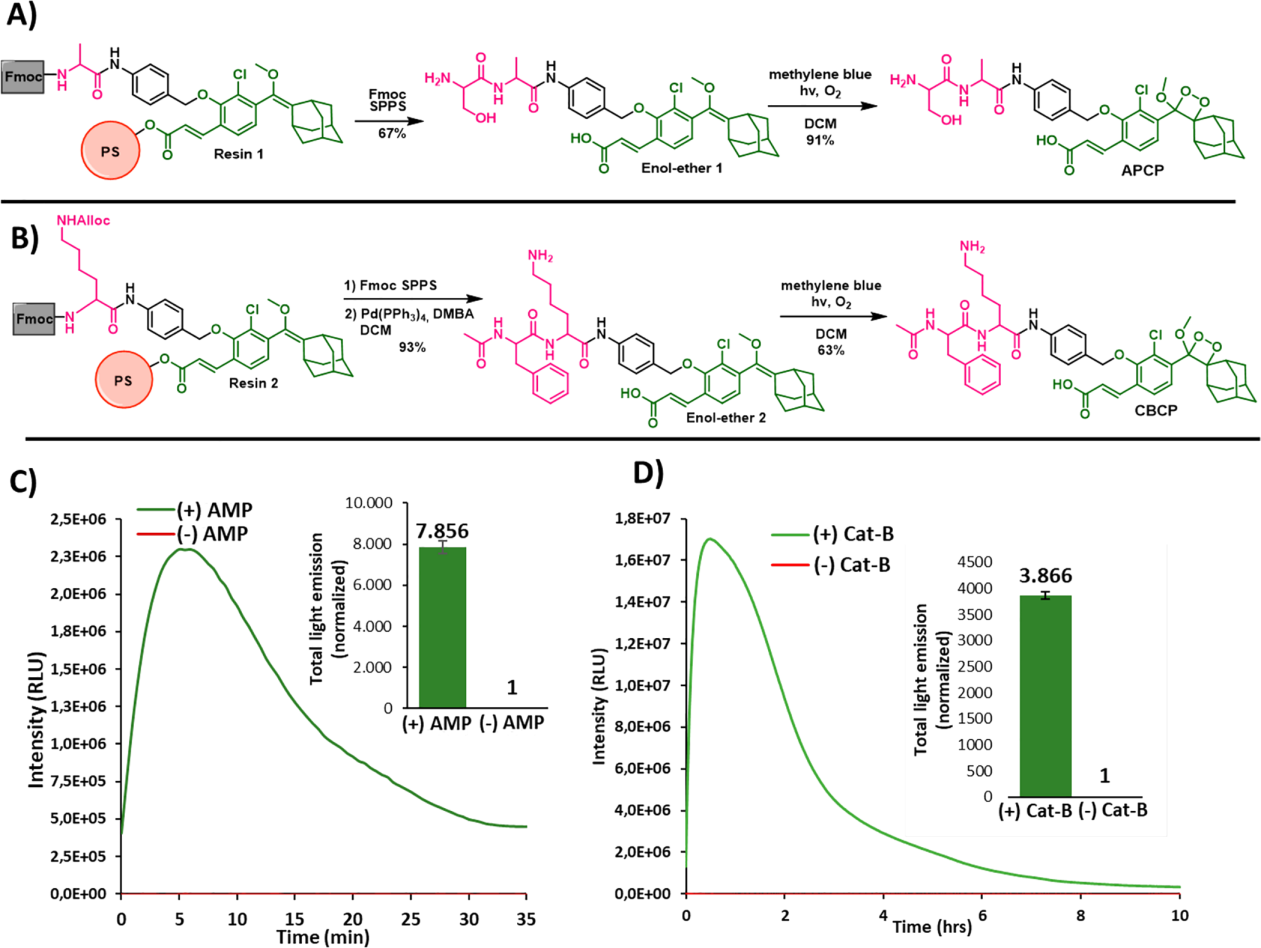
(A) Synthetic route to prepare probe **ACPC**. (B) Synthetic route to prepare probe **CBCP**. (C) Light-emission profile and total light emission of probe **APCP** [10 μM] in the presence and absence of AMP (aminopeptidase M, porcine kidney, [20 nM]), 1% DMSO, in PBS, pH = 7.4. (D) Light-emission profile and total light emission of probe **CBCP** [50 μM] in the presence and absence of Cat-B (cathepsin B, human liver, [1.4 U/mL]), 1% DMSO, in activity buffer (0.1 M PBS [1.37 M], KCl [27 mM], EDTA [1 mM], glutathione [5 mM]).

The light emission profiles of chemiluminescent protease probes **APCP** and **CBCP** were measured in the presence and in the absence of the proteases AMP and Cat-B, respectively. Probe **APCP** in PBS 7.4 showed almost no light emission (Figure 5C). However, in the presence of AMP, a clear increase of signal was detected, displaying a typical chemiluminescent kinetic profile of rise and decay. Remarkably, the total light emitted signal by the probe with AMP was about 7800 times stronger than the signal produced in the absence of the protease.

The light emission profiles of probe **CBCP** obtained in the presence and in the absence of Cat-B are shown in Figure 5D. Likewise, Cat-B was able to activate the probe with an initial sharp increase of the light emission signal, followed by gradual decay. As expected, almost no light emission was observed in the absence of Cat-B, and the signal-to-noise ratio value produced by probe **CBCP** was 3800-fold.

To apply our solid-phase synthetic methodology to a broader scope of protease substrates, we sought to demonstrate a more complex example that incorporates multiple amino acids, including ones that require protecting groups on their side chains. Since the final peptide-enol-ether is cleaved from the 2-chlorotrityl resin by HFIP, we assumed that commercially available trityl-protected amino acids will be suitable for use in our solid-phase synthesis. To validate this hypothesis, we designed a synthetic route to prepare a chemiluminescent probe for the detection of Prostate-Specific Antigen (PSA) (Figure 6A), which was previously prepared by our group via standard synthesis in solution.^27^

**Figure 6:**
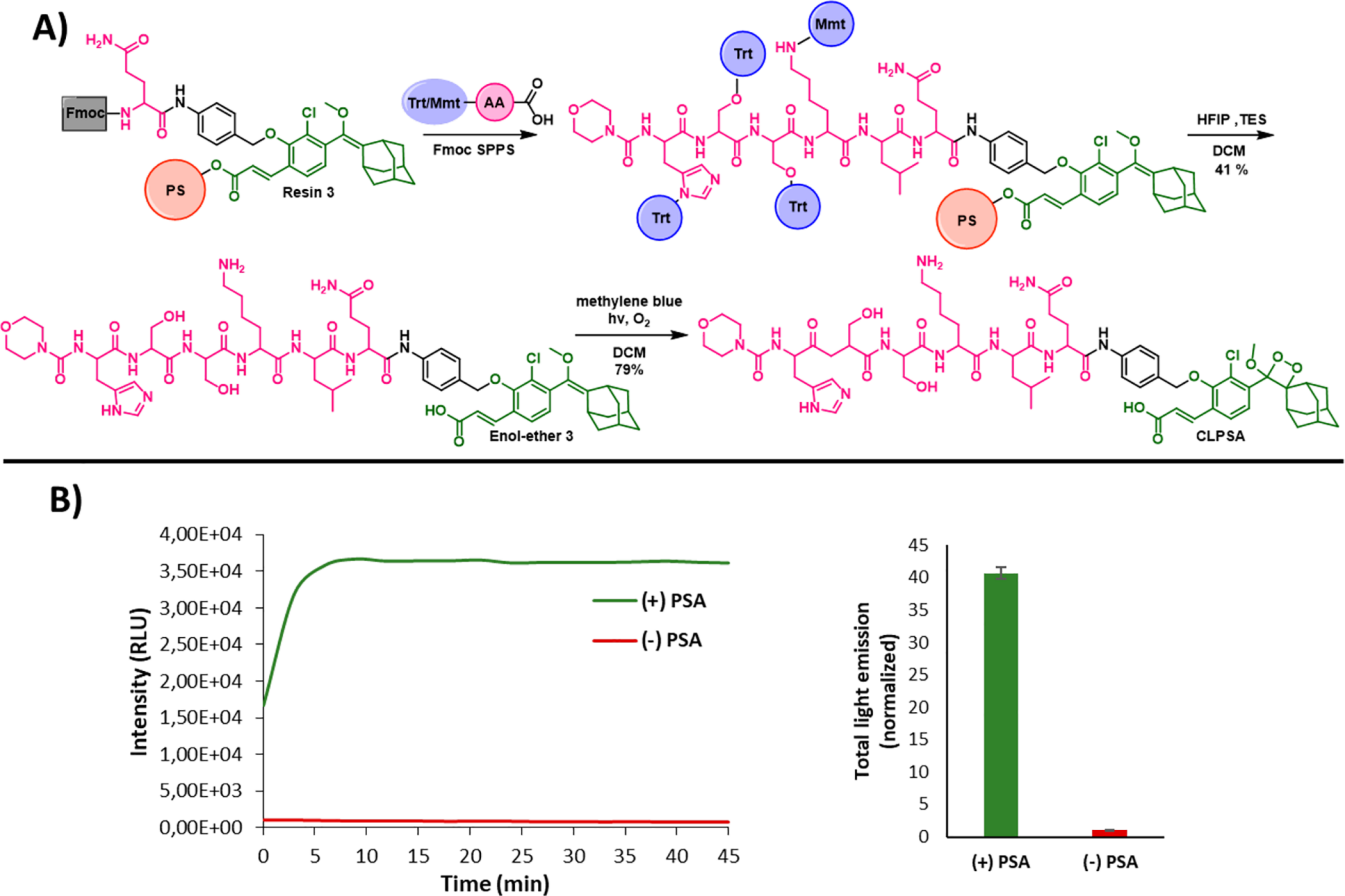
(A) Synthetic route to synthesize **CLPSA** probe using SPPS with trityl protected amino acids. (B) Kinetic profile and total light emission of **CLPSA** [10 μM] in the presence and absence of PSA (prostate specific antigen from human seminal fluid, [10 μg/mL]), 2% DMSO, in PBS, pH = 7.4.

The peptidyl substrate of probe **CLPSA** is composed of the sequence Mu-HSSKLQ. This peptide has four amino acids with side residues that require protecting groups during the solid-phase-synthesis. Ideally, those protecting groups should be removed under the same conditions required for the final cleavage. To test the feasibility of our methodology for preparing probe **CLPSA** by solid-phase-synthesis, we used resin **3** as a starting material and commercially available trityl (Trt) protected His and Ser amino acids. In the case of Lys, we chose the monomethoxytrityl (Mmt) as a protecting group for the ε-amino side chain. The solid-phase-synthesis of probe **CLPSA** enol-ether precursor was achieved through six sequential coupling steps starting from resin **3**. Cleavage by HFIP and subsequent oxidation with singlet oxygen afforded probe **CLPSA** in 41% yield after RP-HPLC purification. Importantly, the four trityl-based protecting groups were efficiently removed from the peptide substrate during the cleavage of the enol-ether precursor from the resin by HFIP.

This example effectively demonstrates that chemiluminescent protease probes can be conveniently synthesized using our solid-phase-synthetic methodology, even when complex peptidyl substrates are required. Although **CLPSA** can be prepared by a solution-phase approach, the solid-phase-synthetic approach is considerably simpler and consumes less time and resources. The light-emission profile of **CLPSA** was then evaluated in the presence and in the absence of PSA. Upon addition of PSA, probe **CLPSA** was able to produce light-emission signal 40 times greater than that observed in PBS 7.4 alone (Figure 6B). The observed steady-state signal is attributed to the relatively low enzymatic activity of PSA.^27^

The method developed in this work, includes an oxidation step, by singlet oxygen, of the peptide-enol-ether intermediate to its corresponding dioxetane. Therefore, an amino acid like methionine (Met) can also be oxidized to its sulfoxide derivative. To clarify the limitations of the synthetic method, we subjected peptides, that include the amino acids Met or Trp, to singlet oxygen conditions. Indeed, oxidation with singlet oxygen of a Met-based conjugate, composed of the sequence, Fmoc-Met-Ala-PABA-Enol-ether, has resulted in the corresponding dioxetane and the sulfoxide derivative for the Met. Oxidation with singlet oxygen of a Trp-based conjugate, composed of the sequence Fmoc-Trp(Boc)-Ala-PABA-Enol-ether, has resulted in the corresponding dioxetane with no side oxidation of the Trp. The Boc protecting group can be removed from the Trp, under mild acidic conditions, after the oxidation of enol-ether to the dioxetane. In addition, subjection of Fmoc-Trp to singlet oxygen has confirmed the stability of this amino acid to the oxidation conditions (see Supporting Information Figures S3–S8).

In summary, we developed a practical and efficient solid-phase synthetic methodology for the general preparation of protease chemiluminescent probes. To validate the applicability of the methodology, we have preloaded 2-chlorotrityl-chloride resin with three chemiluminescent enol-ether precursors, each attached to a different Fmoc-AA. Two of these preloaded resins were used in the preparation of two chemiluminescent probes, by the solid-phase synthesis, with dipeptidyl substrates designed for activation by the AMP and Cat-B proteases. The third preloaded resin, was applied for the synthesis of a chemiluminescent probe aimed for detection of PSA, which is composed of a relatively complex peptidyl substrate. This peptidyl substrate, is comprised of six amino acids, four of which require the use of protecting groups on their side chains. The cleavage conditions of the peptide-enol-ether precursor from the trityl resin by HFIP, were compatible with the removal of the protecting groups and the stability of the enol-ether functionality. We anticipate that the described methodology would be useful for the preparation of vast and diverse peptidyl substrates for chemiluminescent protease probes.

## Supporting Information Available

(Synthetic procedures, characterization data (NMR and MS) for all new compounds and chemiluminescence control experiments PDF)

## References

[ref1] Sanman L. E.; Bogyo M. Activity-Based Profiling of Proteases. Annu. Rev. Biochem. 2014, 83, 249–273. 10.1146/annurev-biochem-060713-035352. 24905783

[ref2] Chung H. K.; Lin M. Z. On the Cutting Edge: Protease-Based Methods for Sensing and Controlling Cell Biology. Nat. Methods 2020, 17, 885–896. 10.1038/s41592-020-0891-z. 32661424

[ref3] Turk B. Targeting Proteases: Successes, Failures and Future Prospects. Nat. Rev. Drug Discovery 2006, 5, 785–799. 10.1038/nrd2092. 16955069

[ref4] Neefjes J.; Dantuma N. P. Fluorescent Probes for Proteolysis: Tools for Drug Discovery. Nat. Rev. Drug Discovery 2004, 3, 58–69. 10.1038/nrd1282. 14708021 PMC7097193

[ref5] Edgington L. E.; Verdoes M.; Bogyo M. Functional Imaging of Proteases: Recent Advances in the Design and Application of Substrate-Based and Activity-Based Probes. Curr. Opin. Chem. Biol. 2011, 15, 798–805. 10.1016/j.cbpa.2011.10.012. 22098719 PMC3237724

[ref6] Rajapaksha A. A.; Fu Y. X.; Guo W. Y.; Liu S. Y.; Li Z. W.; Xiong C. Q.; Yang W. C.; Yang G. F. Review on the Recent Progress in the Development of Fluorescent Probes Targeting Enzymes. Methods Appl. Fluoresc. 2021, 9, 032001 10.1088/2050-6120/abf988. 33873170

[ref7] Zimmerman M.; Ashe B.; Yurewicz E. C.; Patel G. Sensitive Assays for Trypsin, Elastase, and Chymotrypsin Using New Fluorogenic Substrates. Anal. Biochem. 1977, 78, 47–51. 10.1016/0003-2697(77)90006-9. 848756

[ref8] Kisin-Finfer E.; Ferber S.; Blau R.; Satchi-Fainaro R.; Shabat D. Synthesis and Evaluation of New NIR-Fluorescent Probes for Cathepsin B: ICT versus FRET as a Turn-ON Mode-of-Action. Bioorg. Med. Chem. Lett. 2014, 24, 2453–2458. 10.1016/j.bmcl.2014.04.022. 24767838

[ref9] Pryyma A.; Gunasekera S.; Lewin J.; Perrin D. M. Rapid, High-Yielding Solid-Phase Synthesis of Cathepsin-B Cleavable Linkers for Targeted Cancer Therapeutics. Bioconjugate Chem. 2020, 31, 2685–2690. 10.1021/acs.bioconjchem.0c00563. 33274932

[ref10] Behrendt R.; White P.; Offer J. Advances in Fmoc Solid-Phase Peptide Synthesis. J. Pept. Sci. 2016, 22, 4–27. 10.1002/psc.2836. 26785684 PMC4745034

[ref11] Bergmann M.; Zervas L. Über Ein Allgemeines Verfahren Der Peptid-Synthese. Ber. Dtsch. Chem. Ges. B 1932, 65, 1192–1201. 10.1002/cber.19320650722.

[ref12] Merrifield B. Solid Phase Synthesis. Science 1986, 232, 341–348. 10.1126/science.3961484. 3961484

[ref13] Quartararo A. J.; Gates Z. P.; Somsen B. A.; Hartrampf N.; Ye X.; Shimada A.; Kajihara Y.; Ottmann C.; Pentelute B. L. Ultra-Large Chemical Libraries for the Discovery of High-Affinity Peptide Binders. Nat. Commun. 2020, 11, 1–11. 10.1038/s41467-020-16920-3. 32576815 PMC7311396

[ref14] Schneider E. L.; Craik C. S. Positional Scanning Synthetic Combinatorial Libraries for Substrate Profiling. Methods Mol. Biol. 2009, 539, 59–78. 10.1007/978-1-60327-003-8_4. 19377970 PMC3793399

[ref15] Maly D. J.; Leonetti F.; Backes B. J.; Dauber D. S.; Harris J. L.; Craik C. S.; Ellman J. A. Expedient Solid-Phase Synthesis of Fluorogenic Protease Substrates Using the 7-Amino-4-Carbamoylmethylcoumarin (ACC) Fluorophore. J. Org. Chem. 2002, 67, 910–915. 10.1021/jo016140o. 11856036

[ref16] Lentz C. S.; Ordonez A. A.; Kasperkiewicz P.; La Greca F.; O’Donoghue A. J.; Schulze C. J.; Powers J. C.; Craik C. S.; Drag M.; Jain S. K.; Bogyo M. Design of Selective Substrates and Activity-Based Probes for Hydrolase Important for Pathogenesis 1 (HIP1) from Mycobacterium Tuberculosis. ACS Infect. Dis. 2016, 2, 807–815. 10.1021/acsinfecdis.6b00092. 27739665 PMC5109297

[ref17] Choe Y.; Leonetti F.; Greenbaum D. C.; Lecaille F.; Bogyo M.; Brömme D.; Ellman J. A.; Craik C. S. Substrate Profiling of Cysteine Proteases Using a Combinatorial Peptide Library Identifies Functionally Unique Specificities. J. Biol. Chem. 2006, 281, 12824–12832. 10.1074/jbc.M513331200. 16520377

[ref18] Rut W.; Groborz K.; Zhang L.; Sun X.; Zmudzinski M.; Pawlik B.; Wang X.; Jochmans D.; Neyts J.; Młynarski W.; Hilgenfeld R.; Drag M. SARS-CoV-2 M pro Inhibitors and Activity-Based Probes for Patient-Sample Imaging. Nat. Chem. Biol. 2021, 17, 222–228. 10.1038/s41589-020-00689-z. 33093684

[ref19] Gosalia D. N.; Salisbury C. M.; Maly D. J.; Ellman J. A.; Diamond S. L. Profiling Serine Protease Substrate Specificity with Solution Phase Fluorogenic Peptide Microarrays. Proteomics 2005, 5, 1292–1298. 10.1002/pmic.200401011. 15742319

[ref20] Hananya N.; Eldar Boock A.; Bauer C. R.; Satchi-Fainaro R.; Shabat D. Remarkable Enhancement of Chemiluminescent Signal by Dioxetane-Fluorophore Conjugates: Turn-ON Chemiluminescence Probes with Color Modulation for Sensing and Imaging. J. Am. Chem. Soc. 2016, 138, 13438–13446. 10.1021/jacs.6b09173. 27652602

[ref21] Green O.; Eilon T.; Hananya N.; Gutkin S.; Bauer C. R.; Shabat D. Opening a Gateway for Chemiluminescence Cell Imaging: Distinctive Methodology for Design of Bright Chemiluminescent Dioxetane Probes. ACS Cent. Sci. 2017, 3, 349–358. 10.1021/acscentsci.7b00058. 28470053 PMC5408346

[ref22] Hananya N.; Shabat D. A. Glowing Trajectory between Bio- and Chemiluminescence: From Luciferin-Based Probes to Triggerable Dioxetanes. Angew. Chem., Int. Ed. 2017, 56, 16454–16463. 10.1002/anie.201706969. 28967167

[ref23] Gnaim S.; Green O.; Shabat D. The Emergence of Aqueous Chemiluminescence: New Promising Class of Phenoxy 1,2-Dioxetane Luminophores. Chem. Commun. 2018, 54, 2073–2085. 10.1039/C8CC00428E. 29423487

[ref24] Gnaim S.; Scomparin A.; Eldar-Boock A.; Bauer C. R.; Satchi-Fainaro R.; Shabat D. Light Emission Enhancement by Supramolecular Complexation of Chemiluminescence Probes Designed for Bioimaging. Chem. Sci. 2019, 10, 2945–2955. 10.1039/C8SC05174G. 30996873 PMC6427943

[ref25] Babin B. M.; Fernandez-Cuervo G.; Sheng J.; Green O.; Ordonez A. A.; Turner M. L.; Laura J. K.; Sanjay K. J.; Shabat D.; Bogyo M. Chemiluminescent Protease Probe for Rapid, Sensitive, and Inexpensive Detection of Live Mycobacterium tuberculosis. ACS Cent. Sci. 2021, 7, 803–814. 10.1021/acscentsci.0c01345. 34079897 PMC8161474

[ref26] Scott J. I.; Gutkin S.; Green O.; Thompson E. J.; Kitamura T.; Shabat D.; Vendrell M. A. Functional Chemiluminescent Probe for in Vivo Imaging of Natural Killer Cell Activity Against Tumours. Angew. Chem., Int. Ed. 2021, 60, 5699–5703. 10.1002/anie.202011429. PMC7986153 33300671

[ref27] Gutkin S.; Green O.; Raviv G.; Shabat D.; Portnoy O. Powerful Chemiluminescence Probe for Rapid Detection of Prostate Specific Antigen Proteolytic Activity: Forensic Identification of Human Semen. Bioconjugate Chem. 2020, 31, 2488–2493. 10.1021/acs.bioconjchem.0c00500. PMC7677928 33090770

[ref28] Roth-Konforti M.; Green O.; Hupfeld M.; Fieseler L.; Heinrich N.; Ihssen J.; Vorberg R.; Wick L.; Spitz U.; Shabat D. Ultrasensitive Detection of Salmonella and Listeria Monocytogenes by Small-Molecule Chemiluminescence Probes. Angew. Chem. 2019, 131, 10469–10475. 10.1002/ange.201904719. 31233265

[ref29] Son S.; Won M.; Green O.; Hananya N.; Sharma A.; Jeon Y.; Kwak J. H.; Sessler J. L.; Shabat D.; Kim J. S. Chemiluminescent Probe for the In Vitro and In Vivo Imaging of Cancers Over-Expressing NQO1. Angew. Chem., Int. Ed. 2019, 58, 1739–1743. 10.1002/anie.201813032. 30561862

[ref30] Hananya N.; Press O.; Das A.; Scomparin A.; Satchi-Fainaro R.; Sagi I.; Shabat D. Persistent Chemiluminescent Glow of Phenoxy-Dioxetane Luminophore Enables Unique CRET-Based Detection of Proteases. Chem. - Eur. J. 2019, 25, 14679–14687. 10.1002/chem.201903489. 31495978

[ref31] Roth-Konforti M. E.; Bauer C. R.; Shabat D. Unprecedented Sensitivity in a Probe for Monitoring Cathepsin B: Chemiluminescence Microscopy Cell-Imaging of a Natively Expressed Enzyme. Angew. Chem., Int. Ed. 2017, 56, 15633–15638. 10.1002/anie.201709347. 29024539

[ref32] Li H.; Yao Q.; Sun W.; Shao K.; Lu Y.; Chung J.; Kim D.; Fan J.; Long S.; Du J.; Li Y.; Wang J.; Yoon J.; Peng X. Aminopeptidase N Activatable Fluorescent Probe for Tracking Metastatic Cancer and Image-Guided Surgery via in Situ Spraying. J. Am. Chem. Soc. 2020, 142, 6381–6389. 10.1021/jacs.0c01365. 32167306

[ref33] Edgington-Mitchell L. E.; Bogyo M.; Verdoes M. Live Cell Imaging and Profiling of Cysteine Cathepsin Activity Using a Quenched Activity-Based Probe. Methods Mol. Biol. 2017, 1491, 145–159. 10.1007/978-1-4939-6439-0_11. 27778287

[ref34] Suurs F. V.; Qiu S. Q.; Yim J. J.; Schröder C. P.; Timmer-Bosscha H.; Bensen E. S.; Santini J. T.; de Vries E. G. E.; Bogyo M.; van Dam G. M. Fluorescent Image-Guided Surgery in Breast Cancer by Intravenous Application of a Quenched Fluorescence Activity-Based Probe for Cysteine Cathepsins in a Syngeneic Mouse Model. EJNMMI Res. 2020, 10, 1–10. 10.1186/s13550-020-00688-0. 32990883 PMC7524956

[ref35] Sanman L. E.; van der Linden W. A.; Verdoes M.; Bogyo M. Bifunctional Probes of Cathepsin Protease Activity and PH Reveal Alterations in Endolysosomal pH during Bacterial Infection. Cell Chem. Biol. 2016, 23, 793–804. 10.1016/j.chembiol.2016.05.019. 27427229 PMC4982764

