# Supplementary material for: Universal Access to Protease Chemiluminescent Probes through Solid-Phase Synthesis
Source: Bioconjug Chem. 2021 Sep 22;32(10):2134–40. doi: 10.1021/acs.bioconjchem.1c00384 (PMC8532118; doi:10.1021/acs.bioconjchem.1c00384)
Supplement: Supplementary file 1 — bc1c00384_si_001.pdf [file bc1c00384_si_001.pdf]

Universal Access to Protease Chemiluminescent Probes through Solid-Phase Synthesis

Maria Ponomariov[a], Doron Shabat[a]* and Ori Green[a]*

[a]School of Chemistry, Raymond and Beverly Sackler Faculty of Exact Sciences, Tel Aviv University, Tel Aviv 69978 Israel.

Supporting Information

General methods and abbreviations 2

General synthetic schemes 2

Compatibility of Met and Trp towards oxidation with singlet oxygen..3

Experimental procedures for the synthesis of **compounds 1a-3a**..8

Experimental procedures for the synthesis of **compounds 1b-3b**..9

Experimental procedures for the synthesis of **compounds 1c-3c**..10

Experimental procedures for the synthesis of **compounds 1-3**..12

Experimental procedures for the synthesis of **Resin 1-3** (Resin loading)..15

Experimental procedures for the synthesis of **Enol-ether 1-3** (Fmoc SPPS)..16

Experimental procedures for the synthesis of **APCP, CBCP** and **PSACL**..21

NMR spectra..25

General methods

All reactions were carried out at room temperature unless stated otherwise. Chemicals and solvents were either A.R. grade or purified by standard techniques. Thin layer chromatography (TLC): silica gel plates Merck 60 F254: compounds were visualized by irradiation with UV light. Column chromatography (FC): silica gel Merck 60 (particle size 0.040-0.063 mm), eluent given in parentheses. Reverse-phase high pressure liquid chromatography (RP-HPLC): C18 5u, 250x4.6mm, eluent ACN and water with 0.1% TFA, gradient given in parentheses. Preparative RP-HPLC: C18 5u, 250x21mm, eluent ACN and water with 0.1% TFA, gradient given in parentheses. ^{1}H-NMR spectra were recorded using Bruker Avance operated at 400 MHz or 500 MHz. ^{13}C-NMR spectra were recorded using Bruker Avance operated at 100 MHz or 126 MHz. Chemical shifts were reported in ppm on the δ scale relative to a residual solvent (CDCl3: δ = 7.26 for ^{1}H-NMR and 77.16 for ^{13}C-NMR, DMSO-d6: δ = 2.50 for ^{1}H-NMR and 39.52 for 13C-NMR). Mass spectra were measured on Waters Xevo TQD. Chemiluminescence was recorded on Molecular Devices Spectramax i3x.

Abbreviations: AcOH - Acetic acid, **ACN** - Acetonitrile, **DCC** - N,N'-Dicyclohexylcarbodiimide, **DCM** - Dichloromethane, **DIPEA** – Diisopropylethylamine, **DMAP** - 4-Dimethylaminopyridine, **DMBA** – 1,3-Dimethylbarbituric acid, **DMF** - N,N'-Dimethylformamide, **EEDQ** - 2-Ethoxy-1-ethoxycarbonyl-1,2-dihydroquinoline, **EtOAc** – Ethyl acetate, **Hex** - Hexanes, **MeOH** - Methanol, **TFA** - Trifluoroacetic acid, **TMS-Cl** - Trimethylsilyl chloride, **SPPS** – Solid phase peptide synthesis.

General Synthetic schemes

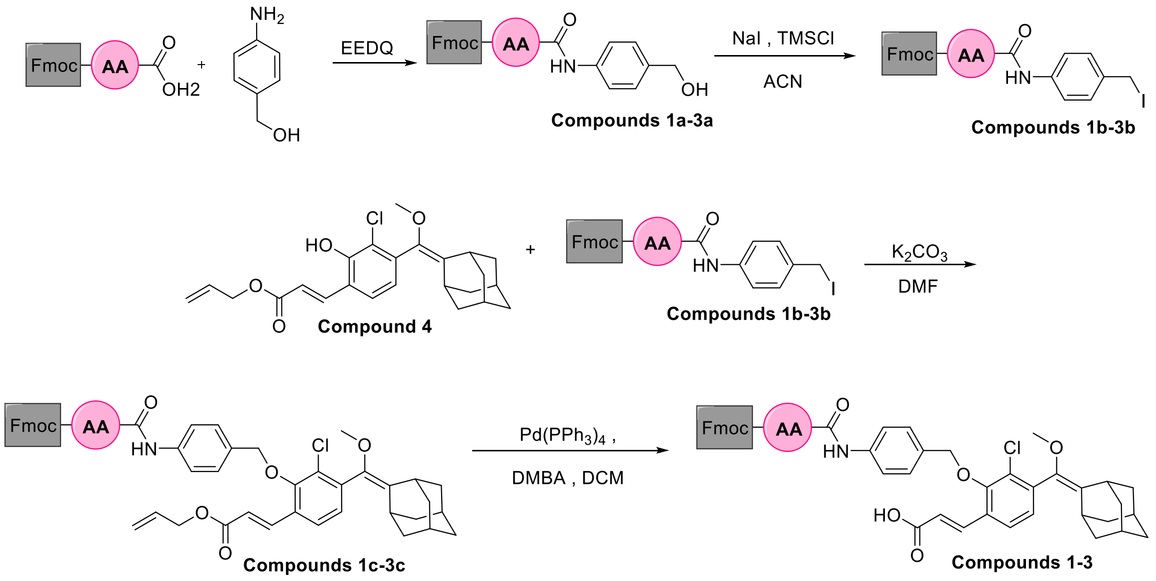

Figure S1. General synthetic scheme for compounds **1**-**3**; Enol-ether protected with Fmoc-AA.

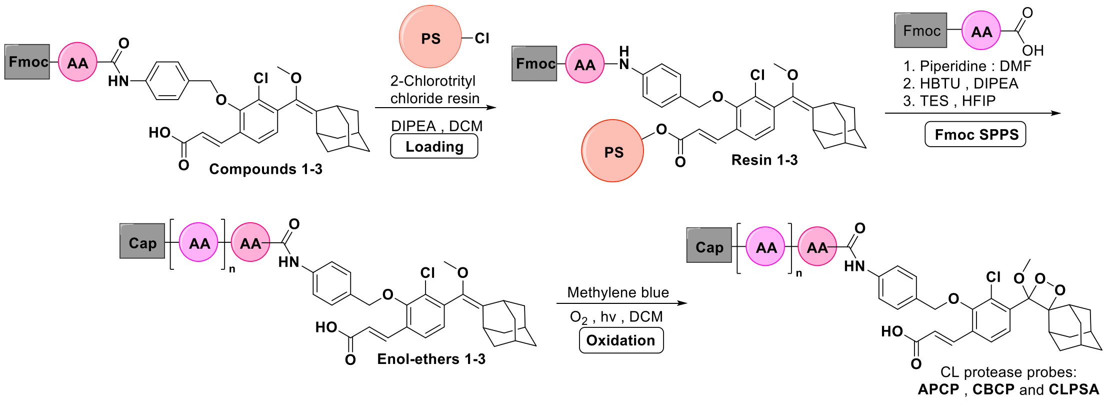

Figure S2. General synthetic scheme for preparing the protease probes **APCP**, **CBCP** and **CLPSA** using SPPS.

Compatibility of Met and Trp towards oxidation with singlet oxygen

The compatibility of the oxidation with the amino acids Met and Trp was tested by performing Fmoc SPPS with a single amino acid (either Fmoc-Met-OH or Fmoc-Trp(Boc)-OH) on **resin 1,** after which the oxidation was performed (as shown in Figure S2). The results are presented below.

Tryptophan:

Coupling:

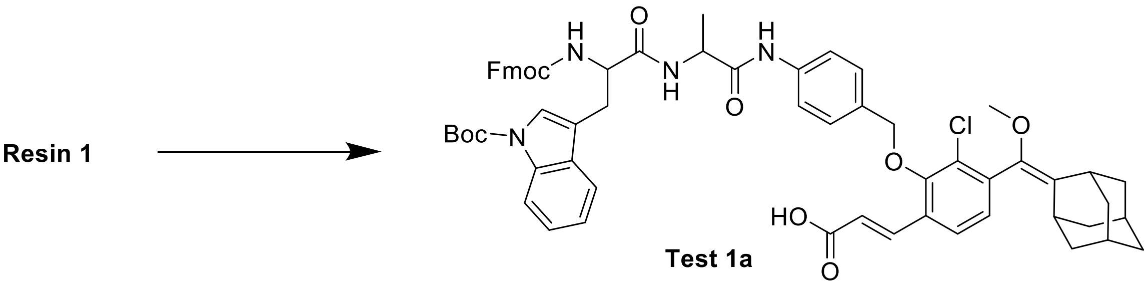

MS (ES+): m/z calculated for $C_{62}H_{63}ClN_4O_{10}$: 1058.4; found: 1081.7 $[M + Na]^+$.

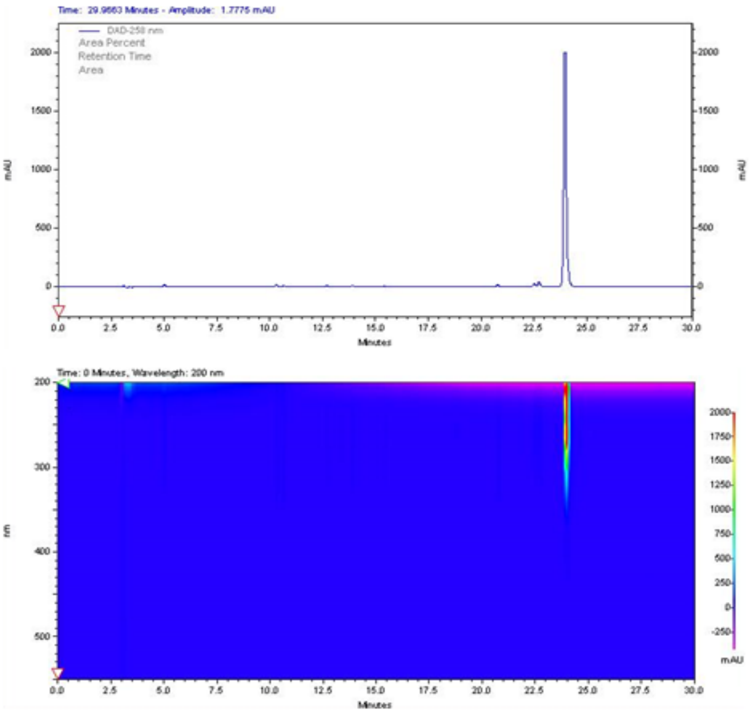

Figure S3. HPLC 2D and 3D chromatogram of **Test-1a** (50-100% ACN:H_2O, RT=24 minutes).

Oxidation:

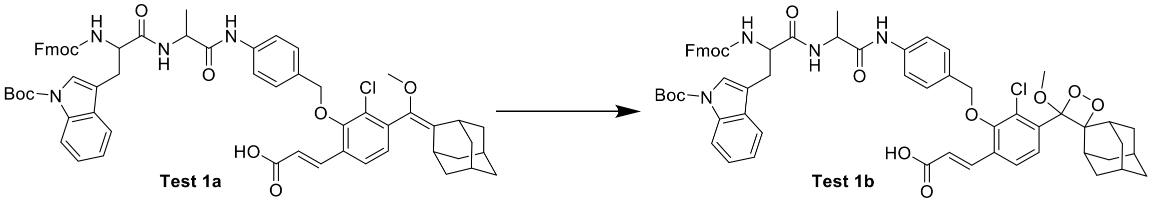

MS (ES+): m/z calculated for $C_{62}H_{63}ClN_4O_{12}$: 1090.4; found: 1113.7 $[M + Na]^+$.

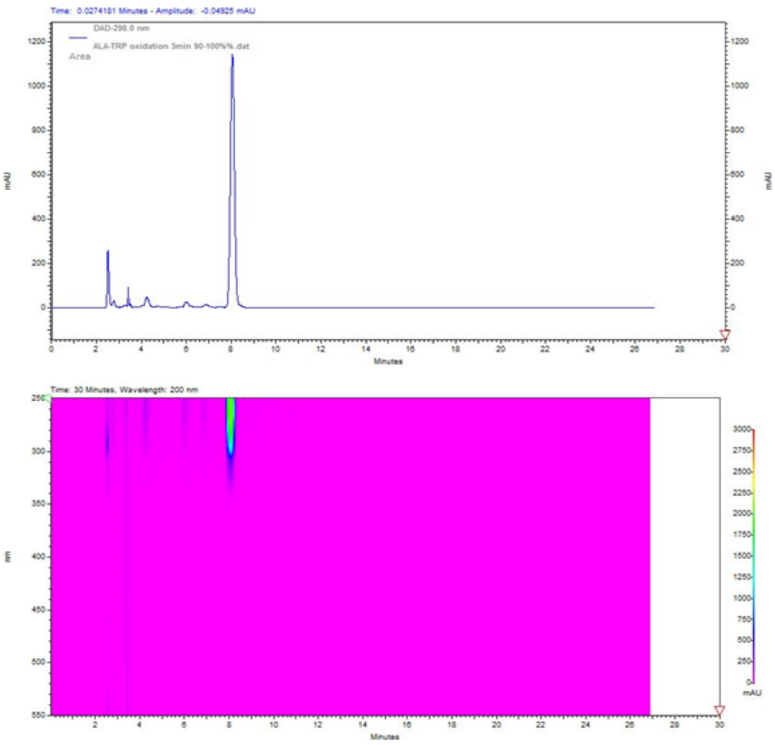

Figure S4. HPLC 2D and 3D chromatogram of **Test-1b** (90-100% ACN:H_2O, RT=8 minutes).

Methionine:

Coupling:

Resin 1

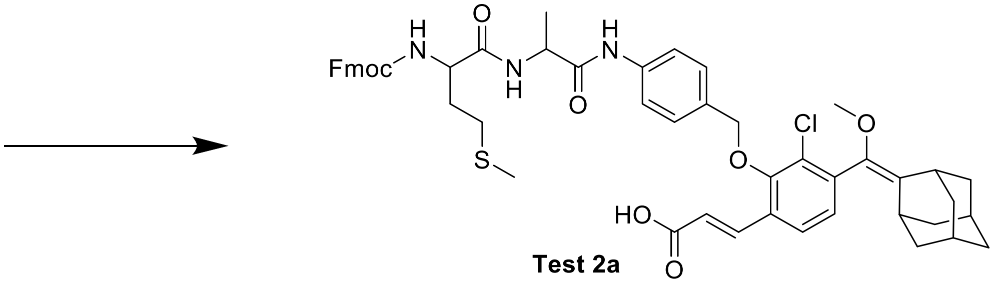

Test 2a

MS (ES+): m/z calculated for $C_{51}H_{54}ClN_3O_8S$: 903.3; found: 926.6 $[M + Na]^+$.

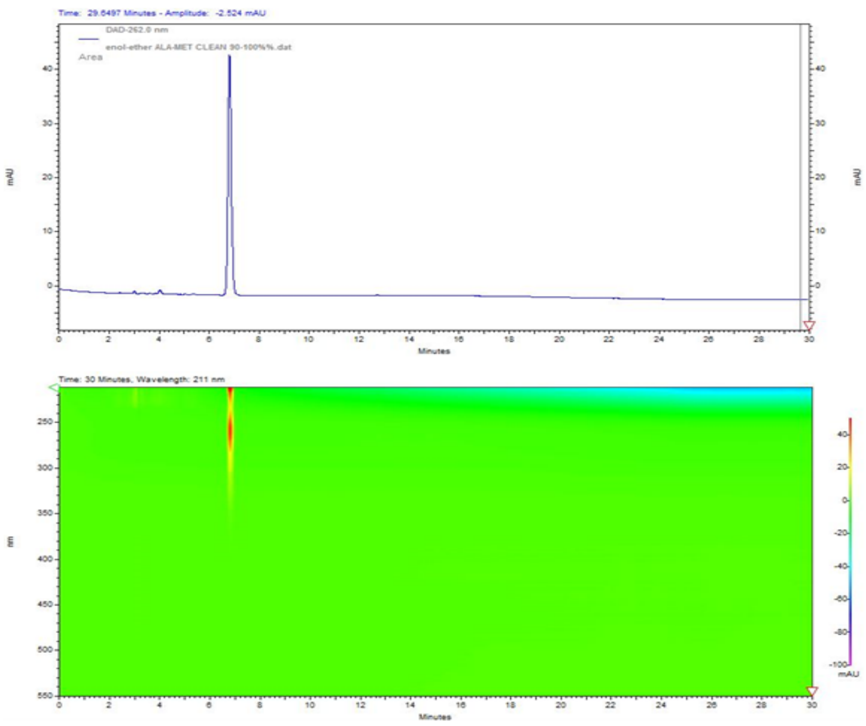

Figure S5. HPLC 2D and 3D chromatogram of **Test-2a** (90-100% ACN:H_2O, RT=6.8 minutes).

Oxidation:

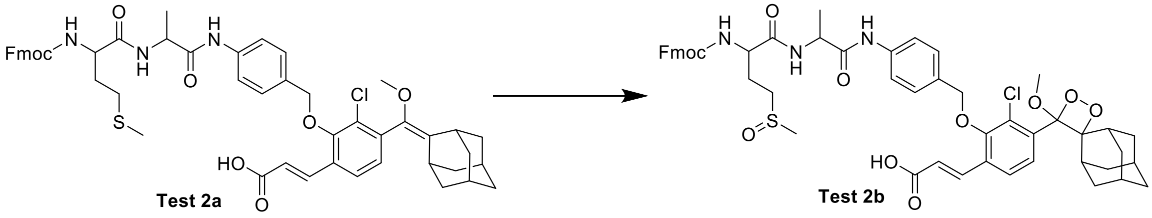

MS (ES+): m/z calculated for $C_{51}H_{54}ClN_3O_{11}S$: 951.3; found: 974.6 $[M + Na]^+$.

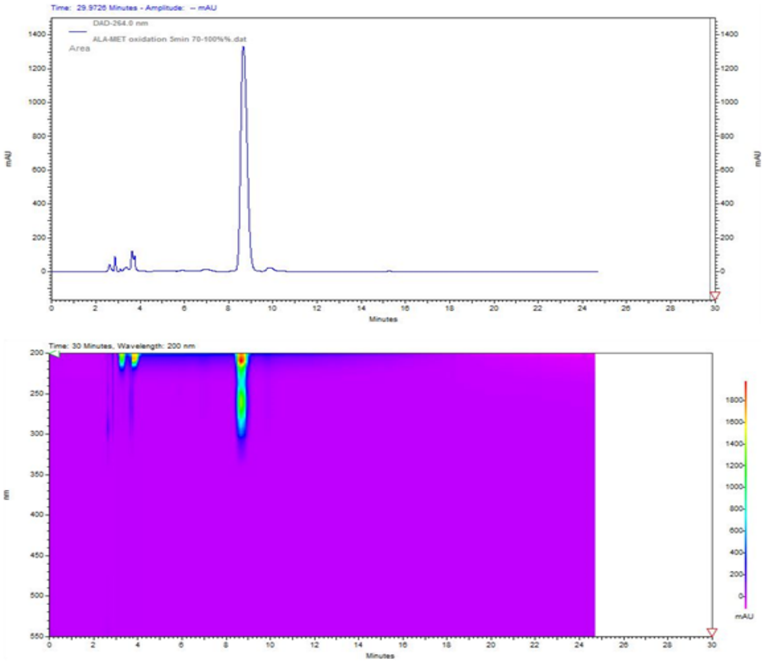

Figure S6. HPLC 2D and 3D chromatogram of **Test-2b** (70-100% ACN:H_2O, RT=8.7 minutes).

In addition to the coupling tests, Fmoc-Trp-OH was exposed to the oxidation conditions to see whether or not the side-chain nitrogen is oxidized by singlet oxygen:

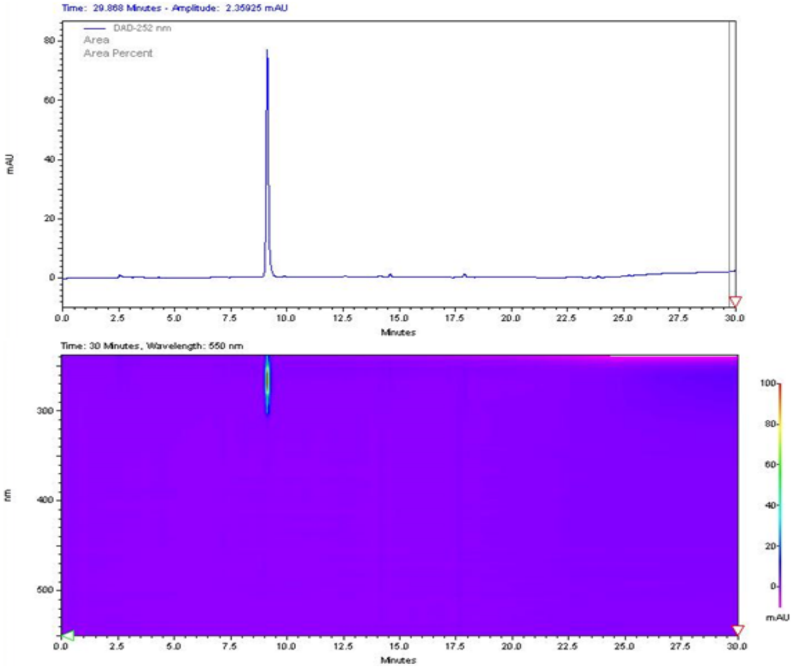

Figure S7. HPLC 2D and 3D chromatogram of Fmoc-Trp-OH (50-100% ACN:H_2O, RT=9.1 minutes).

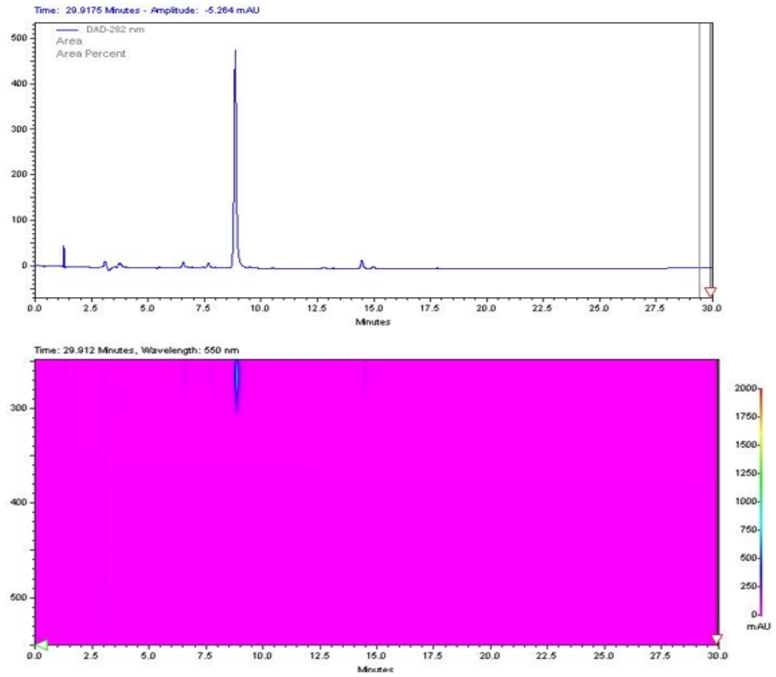

Figure S8. HPLC 2D and 3D chromatogram of Fmoc-Trp-OH after being exposed to singlet oxygen for 5 minutes (50-100% ACN:H_2O, RT=9.1 minutes).

Experimental procedures

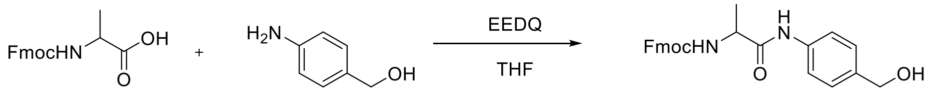

82%

Compound 1a

Compound 1a

Fmoc-Ala-OH (1.05 gr, 3.38 mmol, 1eq), *p*-aminobenzyl alcohol (624 mg, 5.06 mmol, 1.5eq) and EEDQ (1.67 gr, 6.75 mmol, 2eq) were dissolved in THF. The reaction was allowed to stir overnight. Completion of the reaction was confirmed by TLC monitoring (Hex:EtOAc 50:50). Next, the solvent was evaporated under reduced pressure and the crude was triturated with diethyl ether. The solvent was decanted off to afford compound **1a** as a white solid (1.15 gr, 82%). ^{1}H NMR (400 MHz, DMSO) δ 9.96 (s, 1H), 7.86 (d, *J* = 5.6 Hz, 2H), 7.74 (s, 2H), 7.67 (d, *J* = 5.6 Hz, 1H), 7.59 (d, *J* = 6.7 Hz, 2H), 7.40 (s, 2H), 7.35 – 7.20 (m, 4H), 5.12 (s, 1H), 4.46 (s, 2H), 4.36 – 4.11 (m, 4H), 1.33 (d, *J* = 4.5 Hz, 3H). ^{13}C NMR (101 MHz, DMSO) δ 171.50, 155.87, 143.93, 143.83, 140.76, 137.69, 137.45, 127.66, 127.09, 126.94, 125.33, 120.11, 118.98, 65.68, 50.81, 46.70, 18.14. MS (ES+): m/z calculated for $C_{25}H_{24}N_2O_4$: 416.5; found: 417.5 $[M + H]^+$.

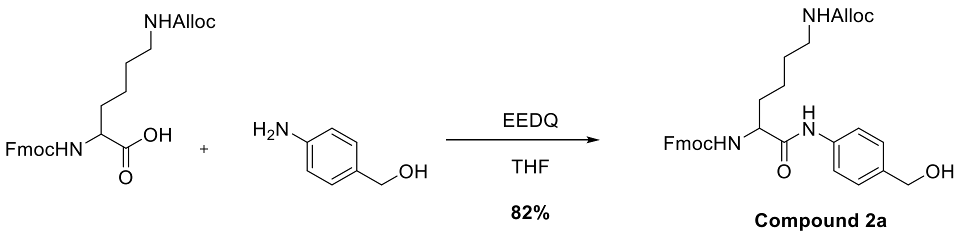

Compound 2a

Fmoc-Lys(Alloc)-OH (420 mg, 0.93 mmol, 1eq), *p*-aminobenzyl alcohol (229 mg, 1.86 mmol, 2eq) and EEDQ (459 mg, 1.86 mmol, 2eq) were dissolved in THF. The reaction was allowed to stir overnight. Completion of the reaction was confirmed by TLC monitoring (Hex:EtOAc 50:50). Next, the solvent was evaporated under reduced pressure and the crude was triturated with diethyl ether. The solvent was decanted off to afford compound **2a** as a white solid (415 mg, 82%). ^{1}H NMR (400 MHz, DMSO) δ 9.98 (s, 1H), 7.85 (s, 2H), 7.73 (s, 2H), 7.66 – 7.57 (m, 3H), 7.39 – 7.18 (m, 6H), 5.87 (s, 1H), 5.24 (d, *J* = 17.1 Hz, 1H), 5.13 (s, 1H), 4.45 (s, 3H), 4.36 – 4.02 (m, 4H), 3.43 (s, 3H), 3.00 (s, 2H), 1.68 (s, 2H), 1.50 – 1.18 (m, 4H). ^{13}C NMR (101 MHz, DMSO) δ 171.09, 156.19, 156.02, 143.85, 140.78, 137.65, 137.52, 133.89, 127.70, 127.12, 126.98, 125.38, 120.14, 119.07, 116.89, 65.74, 64.20, 62.71, 55.49, 46.75, 40.13, 31.65, 29.20, 23.00. MS (ES+): m/z calculated for $C_{32}H_{35}N_{3}O_{6}$: 557.6; found: 558.6 $[M + H]^{+}$.

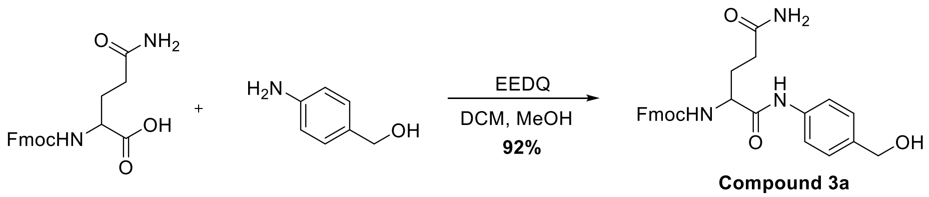

Compound 3a

Compound **3a** was synthesized according to previously reported procedure[2].

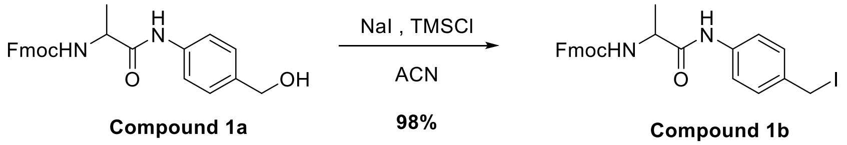

Compound 1b

Benzyl alcohol compound **1a** (1.2 gr, 2.9 mmol, 1eq) and NaI (1.3 gr, 8.6 mmol, 3eq) were dissolved in ACN and cooled to 0°C in an ice bath. TMSCl (1.1 ml, 8.6 mmol, 3eq) was added and the reaction was brought to RT. The reaction was stirred for 30 minutes and monitored by TLC (EtOAc). Upon completion, the solvent was evaporated under reduced pressure. The residue was triturated with diethyl ether and the liquid was decanted off. The product was obtained as an orange solid (1.5 gr, 98%). ^{1}H NMR (500 MHz, $CDCl_3$) δ 8.56 (s, 1H), 7.75 (d, *J* = 7.2 Hz, 1H), 7.56 (t, *J* = 8.2 Hz, 1H), 7.43 (d, *J* = 8.1 Hz, 1H), 7.39 (t, *J* = 7.3 Hz, 1H), 7.27 (d, *J* = 7.3 Hz, 1H), 5.64 (s, 1H), 4.43 (s, 2H), 4.19 (t, *J* = 6.7 Hz, 1H), 1.47 (d, *J* = 5.9 Hz, 1H). ^{13}C NMR (126 MHz, $CDCl_3$) δ 170.29, 143.27, 141.01,

134.94, 129.12, 127.53, 126.85, 124.68, 119.90, 119.75, 67.09, 50.97, 46.77, 30.04, 29.39, 17.93, 5.29. MS (ES+): m/z calculated for $C_{25}H_{23}IN_2O_3$: 526.1; found: 527.4 $[M + H]^+$.

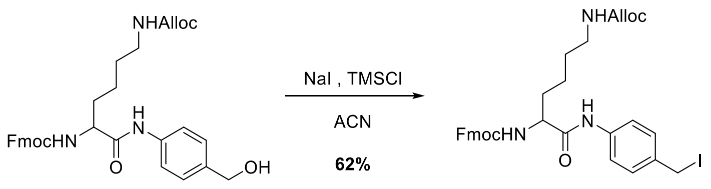

Compound 2a
Compound 2b

Compound 2b

Benzyl alcohol compound **2b** (269 mg, 0.48 mmol, 1eq) and NaI (217 mg, 1.45 mmol, 3eq) were dissolved in ACN and cooled to 0°C in an ice bath. TMSCl (184 µl, 1.45 mmol, 3eq) was added and the reaction was brought to RT. The reaction was stirred for 30 minutes and monitored by TLC (Hex:EtOAc 50:50). Upon completion, the solvent was evaporated under reduced pressure. The residue was triturated with diethyl ether and the liquid was decanted off. The product was obtained as an orange solid (198 mg, 62%). ^{1}H NMR (400 MHz, DMSO) δ 10.08 (s, 1H), 7.89 (d, *J* = 7.2 Hz, 2H), 7.77 – 7.70 (m, 2H), 7.63 (d, *J* = 7.7 Hz, 1H), 7.54 (d, *J* = 8.2 Hz, 1H), 7.37 (m, 6H), 7.19 (s, 1H), 5.95 – 5.82 (m, 1H), 5.25 (d, *J* = 16.9 Hz, 1H), 5.15 (d, *J* = 10.3 Hz, 1H), 4.62 (s, 2H), 4.44 (d, *J* = 3.8 Hz, 2H), 4.28 (d, *J* = 5.1 Hz, 2H), 4.23 (d, *J* = 6.8 Hz, 1H), 4.16 – 4.07 (m, 1H), 2.98 (d, *J* = 4.8 Hz, 2H), 1.65 (s, 2H), 1.52 – 1.12 (m, 7H). ^{13}C NMR (101 MHz, DMSO) δ 171.81, 156.51, 144.39, 141.31, 138.99, 134.95, 134.45, 132.98, 130.04, 128.23, 127.65, 125.91, 120.70, 119.93, 117.44, 66.25, 64.71, 56.03, 47.26, 32.06, 29.73, 23.52, 8.88. MS (ES+): m/z calculated for $C_{32}H_{34}IN_3O_5$: 667.2; found: 668.5 $[M + H]^+$.

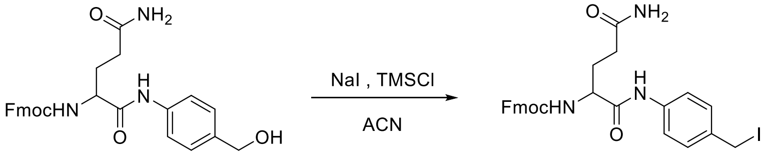

Compound 3a
Compound 3b

Compound 3b

Compound **3b** was synthesized according to previously reported procedure[2].

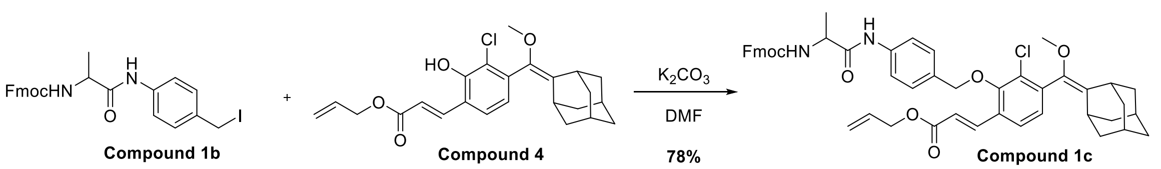

Compound 1b
Compound 4
Compound 1c

Compound 1c

Phenol enol-ether **4**[1] (410 mg, 0.99 mmol, 1eq) was dissolved in anhydrous DMF and cooled to 0°C in an ice bath. Potassium carbonate (164 mg, 1.19 mmol, 1.2eq) was added, and the mixture

stirred for several minutes at 0°C. Next, benzyl iodide compound **1b** (780 mg, 1.48 mmol, 1.5eq) was added and the reaction was brought to RT. The reaction was stirred overnight and monitored by TLC (Hex:EtOAc 50:50). Upon completion, the reaction mixture was diluted with EtOAc and washed with NH_4Cl solution and brine. The organic layer was dried over Na_2SO_4 and evaporated under reduced pressure. The product was purified via column chromatography (Hex:EtOAc 50:50) to afford compound **1c** as a yellow solid (629 mg, 78%). ^{1}H NMR (400 MHz, CDCl$_3$) δ 8.41 (s, 1H), 7.95 (d, *J* = 16.1 Hz, 1H), 7.75 (d, *J* = 7.3 Hz, 2H), 7.56 (d, *J* = 6.5 Hz, 2H), 7.52 (d, *J* = 7.9 Hz, 2H), 7.39 (dd, *J* = 15.6, 7.1 Hz, 4H), 7.31 – 7.25 (m, 2H), 7.07 (d, *J* = 7.9 Hz, 1H), 6.46 (d, *J* = 16.2 Hz, 1H), 6.03 – 5.91 (m, 1H), 5.55 (s, 1H), 5.36 (d, *J* = 17.1 Hz, 1H), 5.27 (d, *J* = 10.4 Hz, 1H), 4.96 (s, 2H), 4.70 (d, *J* = 5.0 Hz, 2H), 4.45 (d, *J* = 6.1 Hz, 2H), 4.21 (t, *J* = 6.4 Hz, 1H), 3.32 (s, 3H), 3.28 (s, 1H), 2.07 (s, 1H), 1.99 – 1.68 (m, 14H), 1.48 (d, *J* = 5.6 Hz, 3H). ^{13}C NMR (101 MHz, CDCl$_3$) δ 170.52, 166.41, 153.81, 143.73, 141.45, 139.56, 139.15, 138.36, 138.10, 132.55, 132.35, 132.11, 129.99, 129.88, 129.80, 127.95, 127.27, 125.21, 125.11, 120.18, 120.10, 119.99, 118.39, 75.85, 67.47, 65.41, 57.38, 47.23, 39.34, 39.19, 38.75, 37.20, 33.09, 29.85, 28.50, 28.35, 18.36. MS (ES+): m/z calculated for $C_{49}H_{49}ClN_2O_7$: 812.3; found: 813.7 $[M + H]^+$.

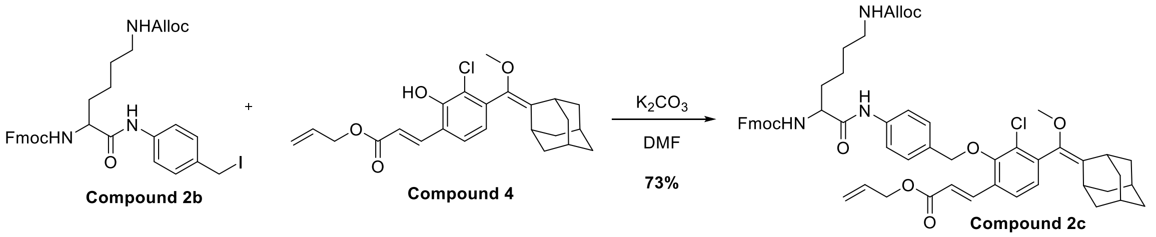

Compound 2c

Phenol enol-ether **4**[1] (106 mg, 0.26 mmol, 1.5eq) was dissolved in anhydrous DMF and cooled to 0°C in an ice bath. Potassium carbonate (47 mg, 0.34 mmol, 2eq) was added, and the mixture stirred for several minutes at 0°C. Next, benzyl iodide compound **2b** (111 mg, 0.17 mmol, 1eq) was added and the reaction was brought to RT. The reaction was stirred overnight and monitored by TLC (Hex:EtOAc 50:50). Upon completion, the reaction mixture was diluted with EtOAc and washed with NH_4Cl solution and brine. The organic layer was dried over Na_2SO_4 and evaporated under reduced pressure. The product was purified via column chromatography (Hex:EtOAc 50:50) to afford compound **2c** as a yellow solid (116 mg, 71%). ^{1}H NMR (400 MHz, CDCl$_3$) δ 8.51 (s, 1H), 7.94 (d, *J* = 16.2 Hz, 1H), 7.75 (d, *J* = 7.3 Hz, 2H), 7.55 (d, *J* = 8.6 Hz, 4H), 7.40 (dt, *J* = 15.3, 7.7 Hz, 5H), 7.31 – 7.23 (m, 2H), 7.07 (d, *J* = 7.9 Hz, 1H), 6.45 (d, *J* = 16.2 Hz, 1H), 6.04 – 5.92 (m, 1H), 5.87 (d, *J* = 5.6 Hz, 1H), 5.71 (s, 1H), 5.36 (d, *J* = 17.2 Hz, 1H), 5.25 (d, *J* = 7.3 Hz, 1H), 5.17 (d, *J* = 10.4 Hz, 1H), 4.95 (s, 3H), 4.70 (d, *J* = 4.9 Hz, 2H), 4.53 (s, 2H), 4.43 (d, *J* = 4.8 Hz, 2H), 4.31 (s, 1H), 4.20 (t, *J* = 6.5 Hz, 1H), 3.32 (s, 3H), 3.28 (s, 1H), 3.19 (s, 2H), 2.07 (s, 1H), 1.97 – 1.67 (m, 14H), 1.42 (d, *J* = 3.9 Hz, 1H), 1.26 (s, 4H). ^{13}C NMR (101 MHz, CDCl$_3$) δ 170.25, 166.42, 156.85, 153.81, 143.79, 141.44, 139.56, 139.14, 138.35, 132.99, 132.54, 132.35, 132.10, 129.99, 129.85, 127.91, 127.25, 125.19, 125.14, 120.15, 120.09, 120.03, 118.40, 117.79, 75.85, 67.35, 65.74, 65.41, 57.38, 55.56, 47.27, 40.14, 39.33, 39.17, 38.74, 37.19, 33.08, 31.65, 31.56, 29.84, 29.46, 28.49, 28.35, 22.41. MS (ES+): m/z calculated for $C_{56}H_{60}ClN_3O_9$: 953.4; found: 954.9 $[M + H]^+$.

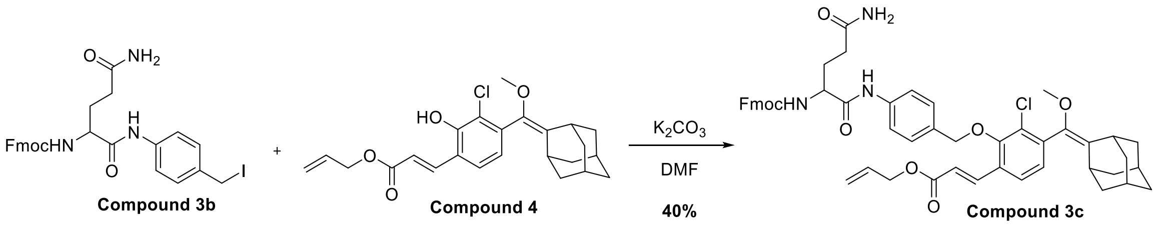

Compound 3c

Phenol enol-ether **4**[1] (95 mg, 0.23 mmol, 1eq) was dissolved in anhydrous DMF and cooled to 0°C in an ice bath. Potassium carbonate (38 mg, 0.28 mmol, 1.2eq) was added, and the mixture stirred for several minutes at 0°C. Next, benzyl iodide compound **3b**[2] (200 mg, 0.34 mmol, 1.5eq) was added and the reaction was brought to RT. The reaction was stirred overnight and monitored by TLC (EtOAc). Upon completion, the reaction mixture was diluted with EtOAc and washed with NH_4Cl and brine. The organic layer was dried over Na_2SO_4 and evaporated under reduced pressure. The product was purified via column chromatography (EtOAc) to afford compound **3c** as a yellow solid (80 mg, 40%). ^{1}H NMR (400 MHz, CDCl$_3$) δ 9.40 (s, 1H), 7.91 (d, *J* = 16.2 Hz, 1H), 7.75 (d, *J* = 7.3 Hz, 2H), 7.58 (d, *J* = 7.3 Hz, 4H), 7.40 (t, *J* = 8.5 Hz, 5H), 7.29 (s, 1H), 7.06 (d, *J* = 7.9 Hz, 1H), 6.42 (d, *J* = 16.2 Hz, 1H), 6.19 (d, *J* = 5.9 Hz, 1H), 6.09 – 5.91 (m, 2H), 5.83 (s, 1H), 5.36 (d, *J* = 17.1 Hz, 1H), 5.28 (d, *J* = 10.9 Hz, 1H), 4.96 (s, 2H), 4.69 (d, *J* = 5.0 Hz, 2H), 4.41 (s, 2H), 4.21 (t, *J* = 6.6 Hz, 1H), 3.32 (s, 3H), 3.28 (s, 1H), 2.52 (s, 1H), 2.41 (s, 1H), 2.23 (s, 1H), 2.06 (s, 1H), 2.00 – 1.62 (m, 14H), 1.42 (d, *J* = 5.5 Hz, 1H). ^{13}C NMR (101 MHz, CDCl$_3$) δ 169.78, 166.44, 153.76, 143.92, 143.76, 141.44, 139.56, 139.19, 138.34, 132.54, 132.34, 131.96, 129.96, 129.88, 127.91, 127.25, 125.23, 125.15, 120.16, 119.93, 118.42, 116.67, 75.92, 67.31, 65.42, 57.37, 54.66, 47.27, 39.17, 38.72, 37.19, 33.08, 29.82, 29.74, 28.49, 28.43, 28.32, 28.24. MS (ES+): m/z calculated for $C_{51}H_{52}ClN_3O_8$: 869.3; found: 870.8 $[M + H]^+$.

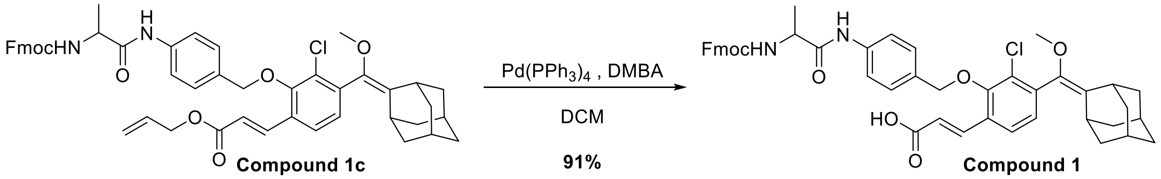

Compound 1

Compound **1c** (527 mg, 0.65 mmol, 1eq) was dissolved in a minimal amount of DCM. DMBA (304 mg, 1.94 mmol, 3eq) and $Pd(PPh_3)_4$ (75 mg, 0.07 mmol, 0.1eq) were added, and the reaction was heated to 35°C. Reaction was stirred for 1 hour and monitored by TLC (Hex:EtOAc 50:50). Upon completion, the reaction mixture was diluted with DCM and washed with NH_4Cl solution and brine. The organic layer was dried over Na_2SO_4 and evaporated under reduced pressure. The product was purified via column chromatography (Hex:EtOAc 50:50) to afford compound **1** as a yellow solid (456 mg, 91%). ^{1}H NMR (400 MHz, CDCl$_3$) δ 9.08 (s, 1H), 7.92 (d, *J* = 16.1 Hz, 1H), 7.81 (s, 1H), 7.70 (d, *J* = 7.1 Hz, 2H), 7.55 (d, *J* = 7.4 Hz, 3H), 7.51 (d, *J* = 7.5 Hz, 1H), 7.41 (d, *J* = 7.8 Hz, 1H), 7.33 (t, *J* = 7.1 Hz, 3H), 7.22 (s, 2H), 7.07 (d, *J* = 7.8 Hz, 1H), 6.39 (d, *J* = 16.4 Hz, 1H), 4.92 (s, 2H), 4.52 (s, 1H), 4.30 (s, 2H), 4.13 (s, 1H), 3.34 (s, 3H), 3.30 (s, 1H), 2.09 (s, 1H), 2.01 – 1.64 (m, 14H), 1.47 (d, *J* = 5.6 Hz, 3H). ^{13}C NMR (101 MHz, CDCl$_3$) δ 171.93, 170.91, 156.88, 153.90, 143.87,

143.65, 141.38, 140.63, 139.53, 138.59, 138.20, 132.74, 132.15, 129.97, 129.83, 129.73, 127.97, 127.90, 127.27, 125.29, 125.22, 120.32, 120.11, 119.58, 75.95, 67.59, 57.43, 51.54, 47.07, 39.34, 38.77, 37.20, 33.12, 29.88, 28.36, 18.59, 1.99. MS (ES+): m/z calculated for $C_{46}H_{45}ClN_2O_7$: 772.3; found: 773.8 $[M + H]^+$.

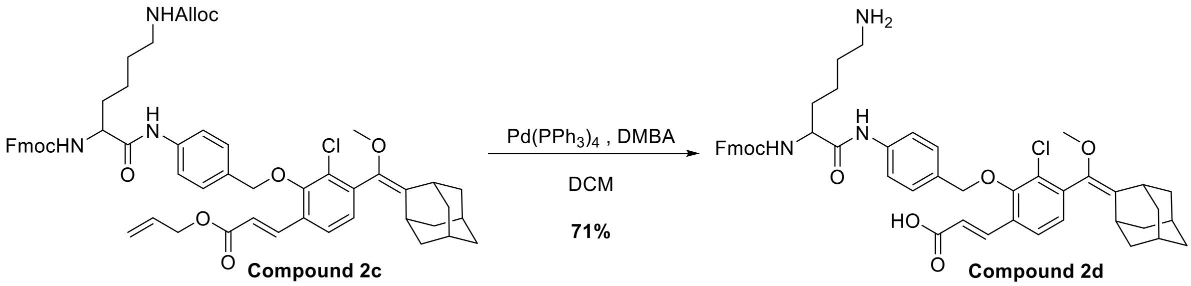

Compound 2d

Compound **2c** (116 mg, 0.12 mmol, 1eq) was dissolved in a minimal amount of DCM. DMBA (114 mg, 0.73 mmol, 6eq) and $Pd(PPh_3)_4$ (14 mg, 0.012 mmol, 0.1eq) were added, and the reaction was heated to 35°C. Reaction was stirred for 1 hour and monitored by RP-HPLC (70-100% ACN). Upon completion, the solvent was evaporated, and the crude was purified via PREP-RP-HPLC (70-100% ACN) to afford product compound **2d** as a white solid (71 mg, 71%). ^{1}H NMR (400 MHz, CDCl$_3$) δ 9.34 (s, 1H), 7.89 – 7.66 (m, 4H), 7.59 (d, *J* = 6.2 Hz, 2H), 7.45 (s, 3H), 7.30 – 7.17 (m, 5H), 7.12 (s, 2H), 6.98 (d, *J* = 7.2 Hz, 1H), 6.47 (s, 1H), 6.26 – 5.84 (m, 4H), 4.82 (s, 2H), 4.23 (s, 3H), 4.02 (s, 1H), 3.28 (s, 4H), 2.92 (s, 2H), 2.06 (s, 1H), 1.98 – 1.54 (m, 14H), 1.48 – 1.30 (m, 2H). ^{13}C NMR (101 MHz, CDCl$_3$) δ 171.48, 169.96, 157.11, 153.73, 143.65, 141.26, 140.22, 139.53, 138.44, 137.91, 132.74, 132.33, 129.96, 129.82, 129.71, 127.93, 127.83, 127.19, 125.11, 120.62, 120.01, 119.45, 75.95, 67.47, 57.38, 46.98, 39.82, 39.18, 38.74, 37.19, 33.10, 31.72, 29.84, 28.46, 28.33, 26.72, 22.33. MS (ES+): m/z calculated for $C_{49}H_{52}ClN_3O_7$: 829.3; found: 830.7 $[M + H]^+$.

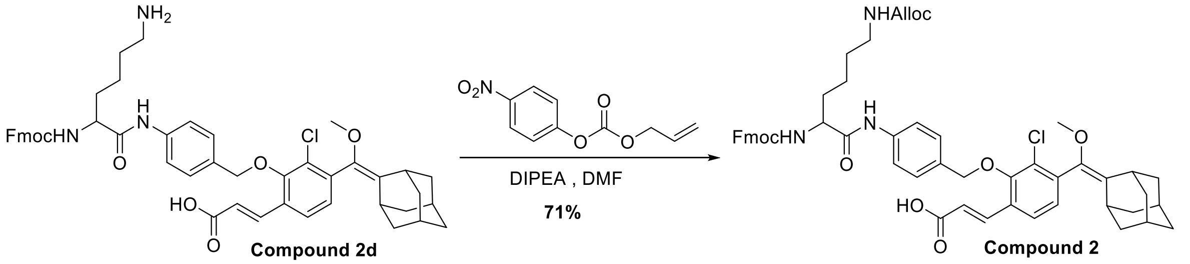

Compound 2

Compounds **2d** (89 mg, 0.107 mmol, 1eq) and allyl-(4-nitrophenyl)-carbonate (34 mg, 0.16 mmol, 1.5eq) were dissolved in a minimal amount of DMF and cooled to 0°C. DIPEA (56 μl, 0.32 mmol, 3eq) was added dropwise and the reaction was brought to RT and monitored by RP-HPLC (70-100% ACN). Upon completion, the crude was purified via PREP-RP-HPLC (70-100% ACN) to afford compound **2** as a white solid (70 mg, 71%). ^{1}H NMR (400 MHz, CDCl$_3$) δ 8.96 (s, 1H), 7.72 (d, *J* =

5.6 Hz, 2H), 7.54 (s, 4H), 7.44 – 7.35 (m, 5H), 7.25 (d, *J* = 6.6 Hz, 2H), 7.07 (d, *J* = 6.2 Hz, 1H), 5.44 – 5.09 (m, 7H), 4.95 (s, 2H), 4.66 – 4.44 (m, 3H), 4.35 (s, 3H), 4.15 (s, 1H), 3.34 (s, 3H), 3.29 (s, 1H), 3.16 (s, 2H), 2.09 (s, 1H), 2.02 – 1.63 (m, 14H), 1.49 (d, *J* = 30.4 Hz, 5H). ^{13}C NMR (101 MHz, $CDCl_3$) δ 171.22, 170.19, 157.01, 143.83, 143.71, 141.40, 140.51, 139.54, 138.08, 132.88, 132.72, 129.94, 127.89, 127.24, 125.19, 120.38, 120.10, 119.39, 117.88, 76.10, 67.52, 66.37, 65.84, 65.46, 57.41, 55.73, 47.15, 40.31, 39.33, 39.18, 38.78, 37.19, 33.12, 31.86, 29.87, 29.42, 29.34, 29.22, 29.18, 28.49, 28.34, 22.51. MS (ES+): m/z calculated for $C_{53}H_{56}ClN_3O_9$: 913.4; found: 914.8 $[M + H]^+$.

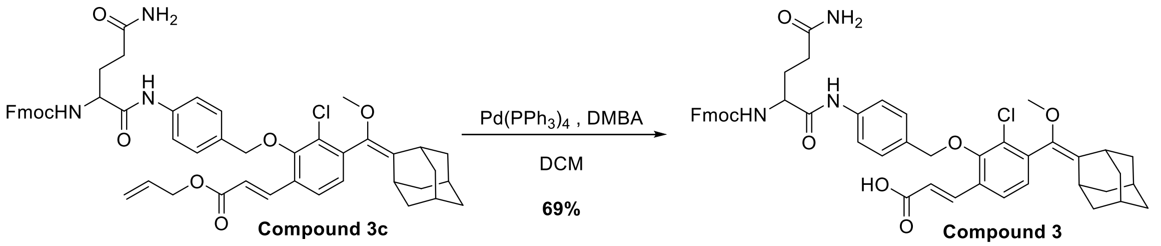

Compound 3

Compound **3c** (80 mg, 0.09 mmol, 1eq) was dissolved in a minimal amount of DCM. DMBA (43 mg, 0.28 mmol, 3eq) and $Pd(PPh_3)_4$ (10.6 mg, 0.009 mmol, 0.1eq) were added, and the reaction was heated to 35°C. Reaction was stirred for 1 hour and monitored by RP-HPLC (70-100% ACN). Upon completion, the solvent was evaporated and the crude was purified via PREP-RP-HPLC (70-100% ACN) to afford Compound **3** as a white solid (51 mg, 69%). ^{1}H NMR (400 MHz, $CDCl_3$) δ 9.52 (s, 1H), 7.71 (d, *J* = 16.0 Hz, 1H), 7.64 (d, *J* = 7.0 Hz, 2H), 7.50 (dd, *J* = 20.2, 7.4 Hz, 4H), 7.32 (d, *J* = 7.9 Hz, 1H), 7.30 – 7.20 (m, 4H), 7.17 (s, 2H), 7.02 (d, *J* = 7.8 Hz, 1H), 6.90 (s, 1H), 6.77 (s, 1H), 6.69 (s, 1H), 6.21 (d, *J* = 16.1 Hz, 1H), 4.87 (s, 2H), 4.49 (s, 1H), 4.26 (s, 2H), 4.07 (s, 1H), 3.31 (s, 3H), 3.28 (s, 1H), 2.39 (s, 1H), 2.23 (s, 1H), 2.08 (s, 2H), 2.03 – 1.62 (m, 14H). ^{13}C NMR (101 MHz, $CDCl_3$) δ 177.22, 170.72, 170.05, 157.08, 153.85, 143.83, 143.64, 141.31, 140.17, 140.07, 139.57, 138.37, 138.19, 132.64, 132.12, 130.13, 130.02, 129.79, 127.85, 127.22, 125.21, 125.04, 120.34, 120.06, 119.59, 116.55, 76.18, 67.46, 57.40, 55.09, 47.08, 39.31, 38.80, 37.21, 33.11, 29.86, 28.35. MS (ES+): m/z calculated for $C_{48}H_{48}ClN_3O_8$: 829.3; found: 830.8 $[M + H]^+$.

General resin loading procedure

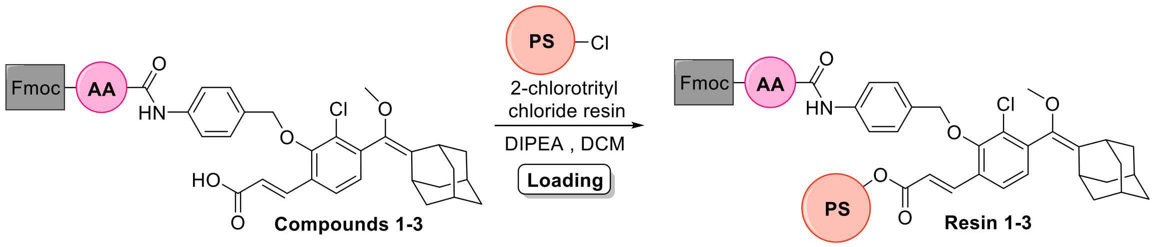

The resin 2-chlorotrityl chloride (5eq) was swelled by swirling in DCM for 2 hours. Next, a solution of ccompounds **1-3** (1eq) and DIPEA (6eq) in DCM were added to the resin and the reaction was swirled overnight. Then, the loading solution was removed, and the resin was washed with DCM three times. Next, the capping solution (9 ml DCM, 1 ml MeOH and 0.5 ml DIPEA) was added to the resin and swirled for 1 hour. Then, the capping solution was removed, and the resin was washed with DCM three times to give **Resin 1-3**.

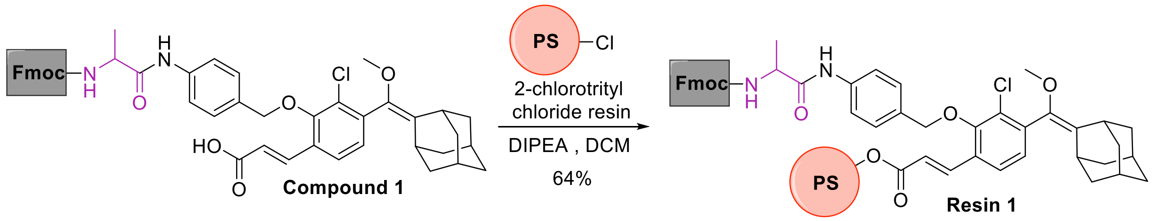

Resin 1

Resin 1 was prepared according to the general loading procedure on 0.65 mmol scale, 64% yield.

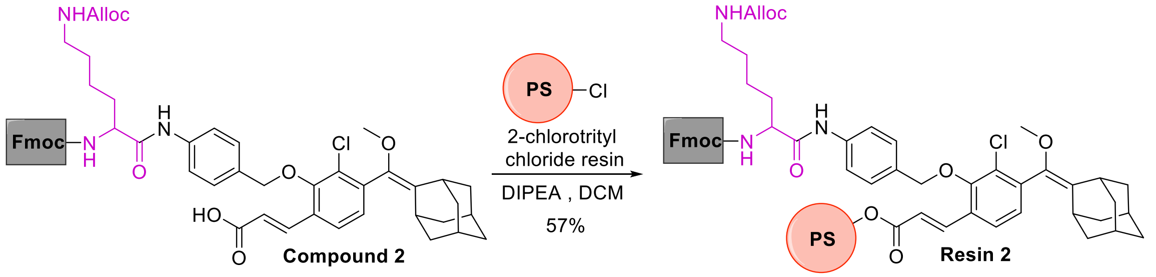

Resin 2

Resin 2 was prepared according to the general loading procedure on 0.08 mmol scale, 57% yield.

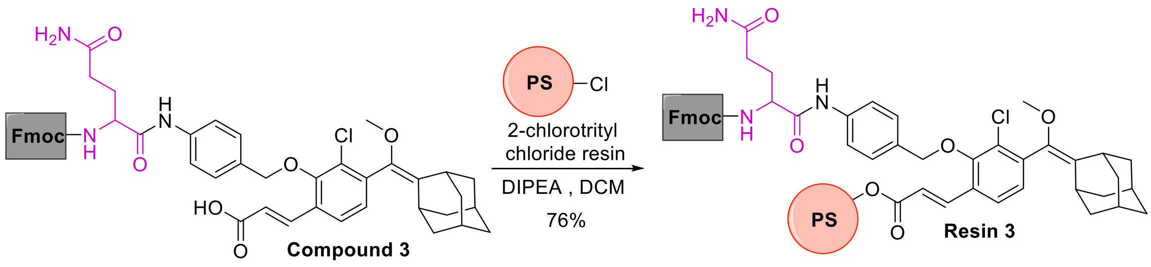

Resin 3

Resin 3 was prepared according to the general loading procedure on 0.15 mmol scale, 76% yield.

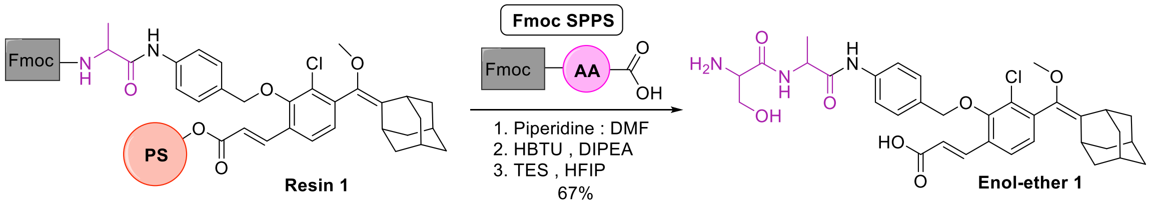

Enol-ether 1

Enol-ether 1 was synthesized via Fmoc-Solid Phase Peptide Synthesis. **Resin 1** (250 mg, 0.046 mmol, 1eq) was swirled in DCM for 30 min. Fmoc deprotection was conducted with 20% piperidine in DMF (15 minutes) followed by coupling of Fmoc-Ser(Trt)-OH (105 mg, 0.184 mmol, 4eq) using HBTU (70 mg, 0.184 mmol, 4eq) and DIPEA (48 µl, 0.276 mmol, 6eq) in DMF (30 minutes).Next, Fmoc deprotection of the serine was completed and to the resin was added the cleavage cocktail (HFIP/DCM/TES [50:45:5]) followed by swirling for 2 hours (also provided Trt deprotection). After filtration, the solvent was evaporated under reduced pressure, and the crude was triturated using diethyl ether followed by filtration to afford the title compound **Enol-ether 1** as a white solid (19.6 mg, 67%). MS (ES+): m/z calculated for $C_{34}H_{40}ClN_3O_7$: 637.3; found: 638.5 $[M+H]^+$.

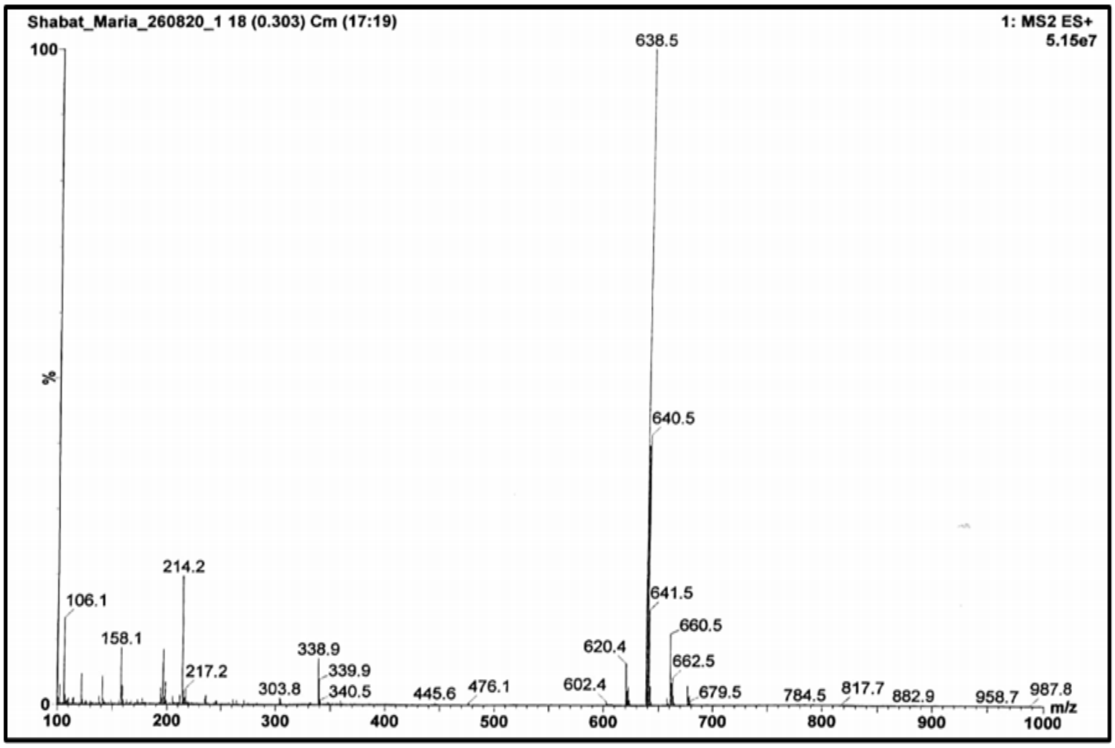

Figure S9. MS(+) spectra of **Enol-ether 1**.

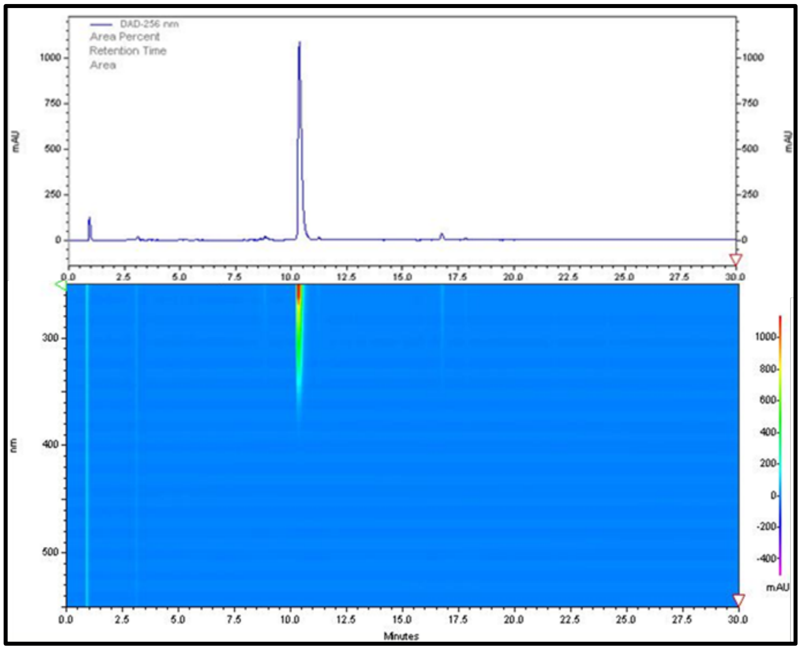

Figure S10. HPLC 2D and 3D chromatogram of **Enol-ether 1** (50-100% ACN:H_2O, RT=10.2 minutes).

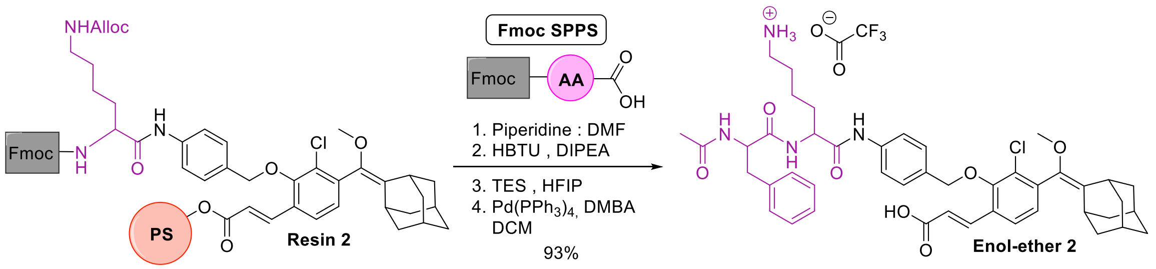

Enol-ether 2

Enol-ether 2 was synthesized via Fmoc-Solid Phase Peptide Synthesis. **Resin 2** (225 mg, 0.025 mmol, 1 eq) was swirled in DCM for 30 min. Fmoc deprotection was conducted with 20% piperidine in DMF (15 minutes) followed by coupling of the Fmoc-Phe-OH (39 mg, 0.1 mmol, 4 eq) using HBTU (38 mg, 0.1 mmol, 4 eq) and DIPEA (26 µl, 0.15 mmol, 6eq) in DMF (30 minutes). Next, Fmoc deprotection of the phenylalanine was completed and the resin was subjected to caping using Ac_2O (24 µL, 0.25 mmol, 10 eq) and DIPEA (65 µL, 0.38 mmol, 15 eq). Next, the resin was added the cleavage cocktail (HFIP/DCM/TES [50:45:5]) and swirled for 1 hour. After filtration, the solvent was evaporated under reduced pressure, and the crude was triturated using diethyl ether followed by filtration of the solid. The crude was reacted without further purification with DMBA (12 mg, 0.075 mmol, 3eq) and $Pd(PPh_3)_4$ (3 mg, 0.0025 mmol, 0.1eq) in DCM and monitored by HPLC (50-100% ACN). Upon completion, the solvent was evaporated, and the compound was purified via PREP-RP-HPLC (50-100% ACN) to afford **Enol-ether 2** as a white solid (19 mg, 93% yield). MS (ES+): m/z calculated for $C_{45}H_{53}ClN_4O_7$: 796.4; found: 797.8 $[M+H]^+$.

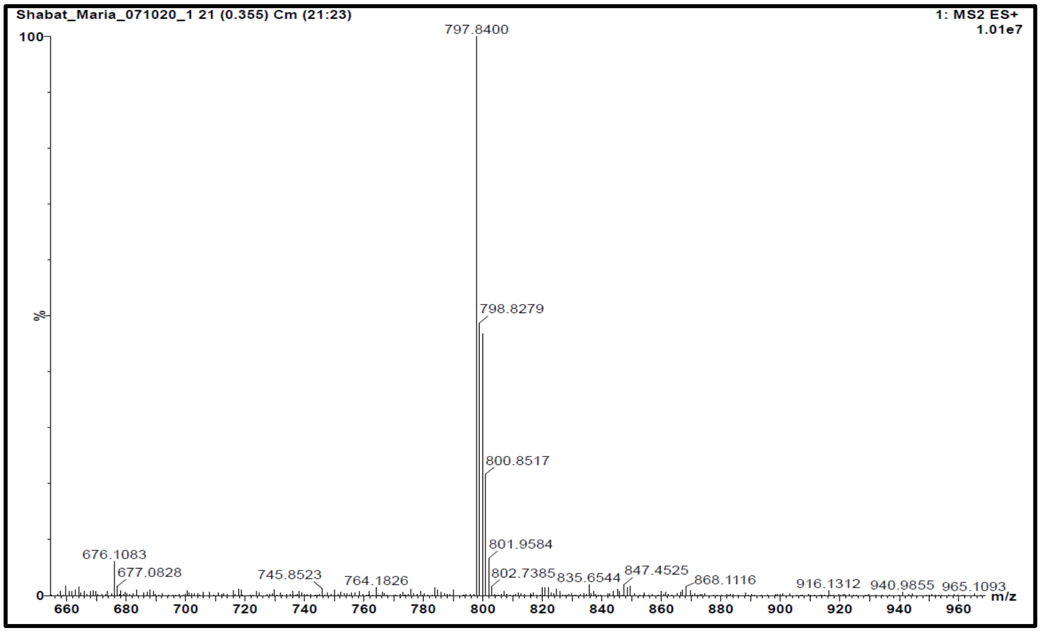

Figure S11. MS(+) spectra of **Enol-ether 2**.

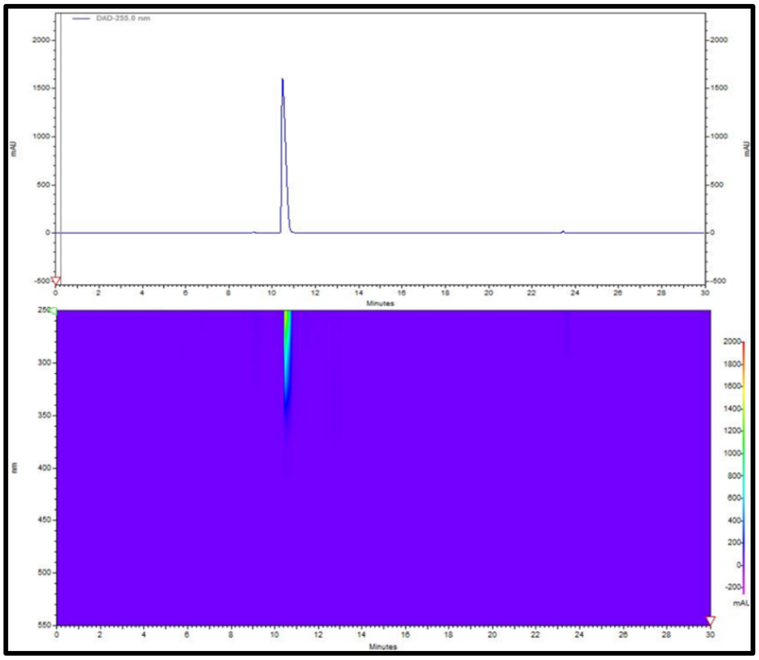

Figure S12. HPLC 2D and 3D chromatogram of **Enol-ether 2** (50-100% ACN:H_2O, RT=10.5 minutes).

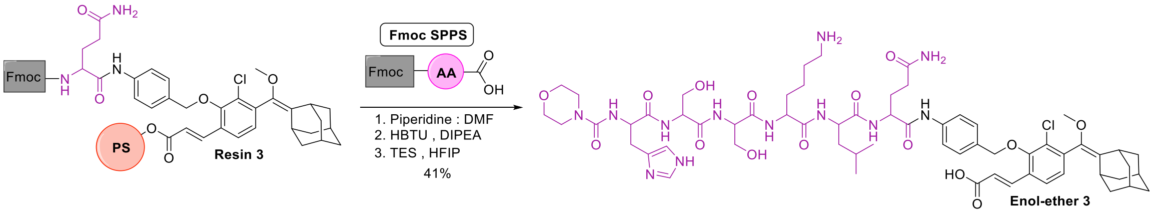

Enol-ether 3

Enol-ether 3 was synthesized via Fmoc-Solid Phase Peptide Synthesis. **Resin 3** (215 mg, 0.063 mmol, 1eq) was swirled in DCM for 30 min. Fmoc deprotection was done with 20% piperidine in DMF (15 minutes) followed by coupling of the next amino acid (4 eq) using HBTU (4 eq) and DIPEA (6 eq) in DMF (30 minutes). These two steps were repeated until the sequence is completed. After Fmoc deprotection of the histidine was completed, the peptide was capped with 4-morpholinecarbonyl chloride (110 µl, 0.95 mmol, 15eq) and DIPEA (329 µl, 1.89 mmol, 30eq) in DMF. Finally, Next, the resin was added the cleavage cocktail (HFIP/DCM/TES [50:45:5]), which also provided Mmt and Trt deprotection after swirling for 2 hours. After filtration, the solvent was evaporated under reduced pressure, and the crude was triturated using diethyl ether followed by filtration to afford the title compound **Enol-ether 3** as a white solid (32.3 mg, 41%). **MS** (ES-): m/z calculated for $C_{62}H_{85}ClN_{12}O_{15}$: 1272.6; found: 1272.3. **MS** (ES+): m/z calculated for $C_{62}H_{85}ClN_{12}O_{15}$: 1272.6; found: 637.9.[(M+2H)/2z]$^+$

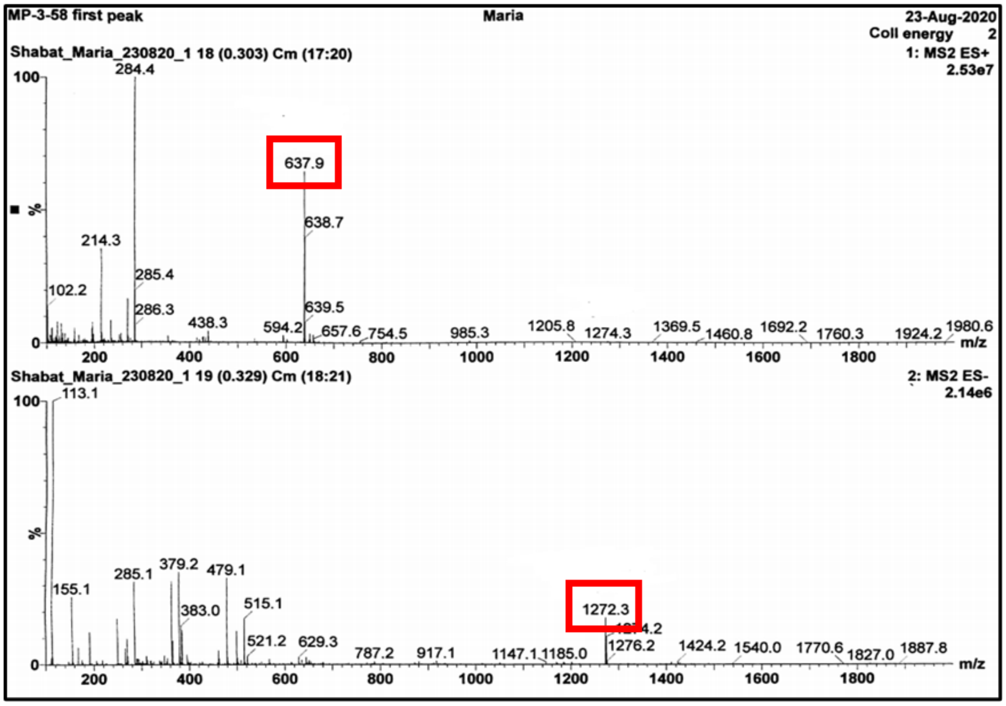

Figure S13. MS(+) spectra of **Enol-ether 3**.

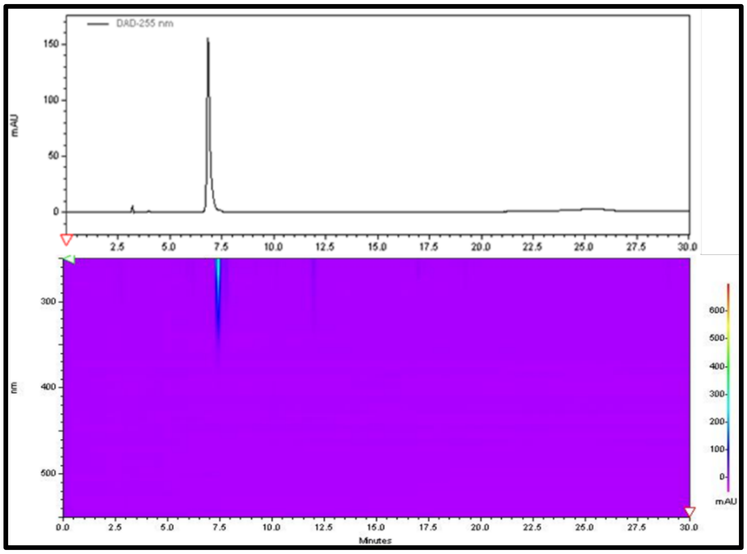

Figure S14. HPLC 2D and 3D chromatogram of **Enol-ether 3** (50-100% ACN:H_2O, RT=7.4 minutes).

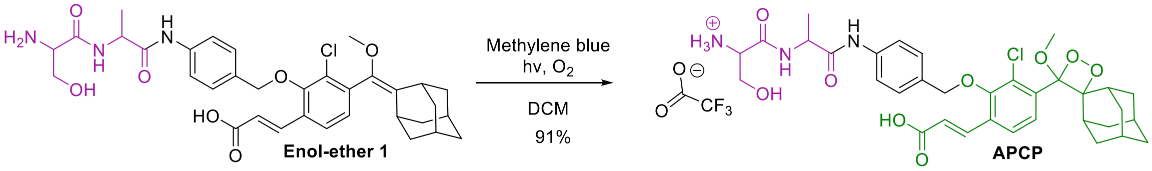

APCP

Enol-ether 1 (9.8 mg, 0.015 mmol) and few milligrams of methylene blue were dissolved in 10 ml of DMF. Oxygen was bubbled through the solution while irradiating with yellow light for 10 minutes. The reaction was monitored by RP-HPLC. After completion, the reaction mixture was purified by preparative RP-HPLC (50-100% ACN). The product **APCP** was obtained as a white solid (9.4 mg, 91%). MS (ES+): m/z calculated for $C_{34}H_{40}ClN_3O_9$: 669.2; found: 670.5 $[M+H]^+$.

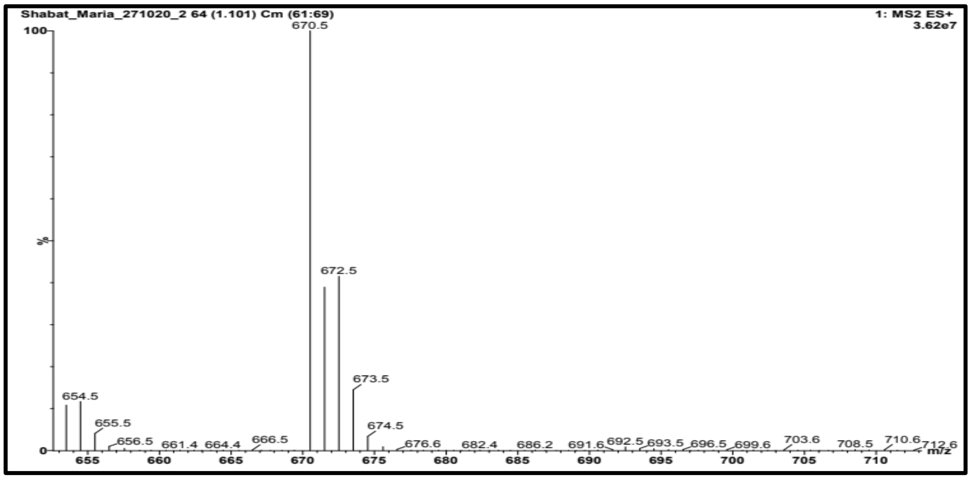

Figure S15. MS(+) spectra of **APCP**.

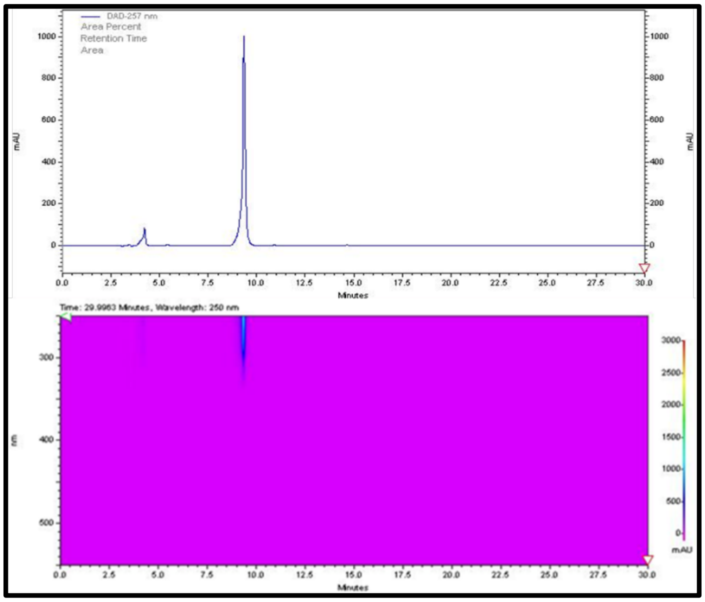

Figure S16. HPLC 2D and 3D chromatogram of **APCP** (50-100% ACN:H_2O, RT=9.3 minutes).

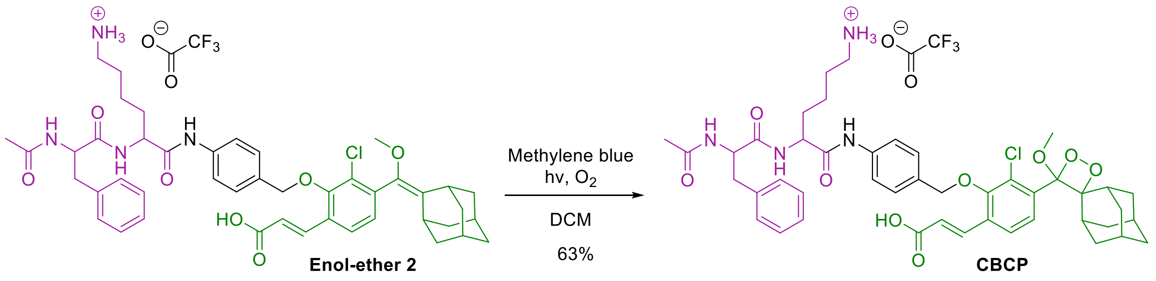

CBCP

Enol-ether 2 (9.1 mg, 0.011 mmol) and few milligrams of methylene blue were dissolved in 2 ml of DMF. Oxygen was bubbled through the solution while irradiating with yellow light for 5 minutes. The reaction was monitored by RP-HPLC. Upon completion, the reaction mixture was purified by preparative RP-HPLC (50-100% ACN). The product **CBCP** was obtained as a white solid (5.94 mg, 63%). MS (ES+): m/z calculated for $C_{45}H_{53}ClN_4O_9$: 828.4; found: 829.7 $[M+H]^+$.

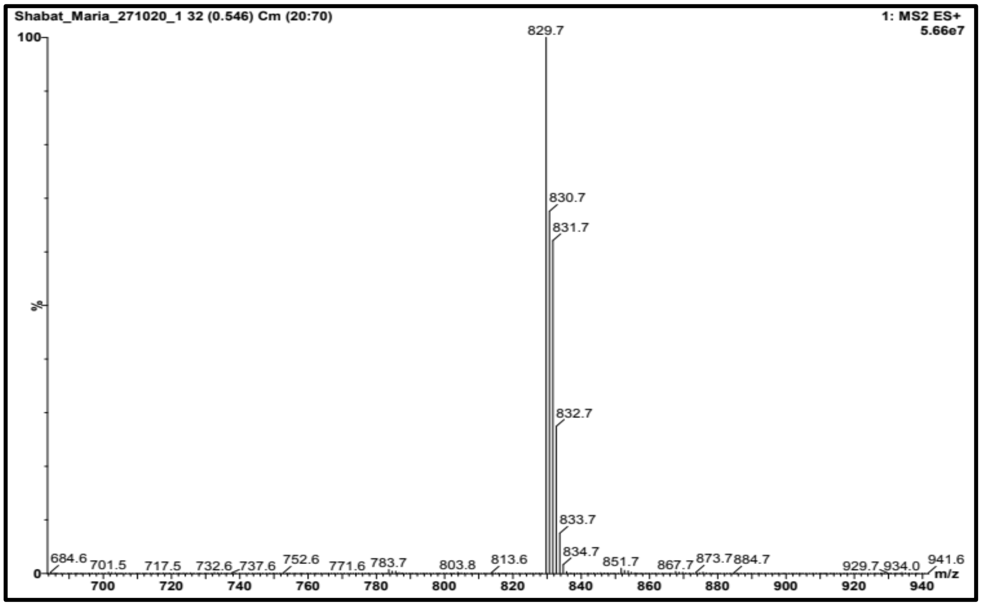

Figure S17. MS(+) spectra of **CBCP**.

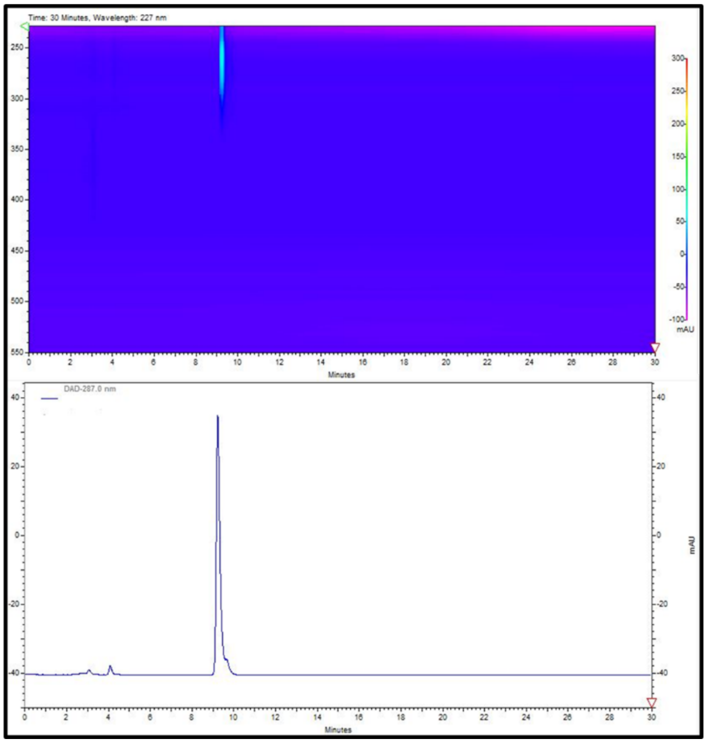

Figure S18. HPLC 2D and 3D chromatogram of **CBCP** (50-100% ACN:H_2O, RT=14.6 minutes).

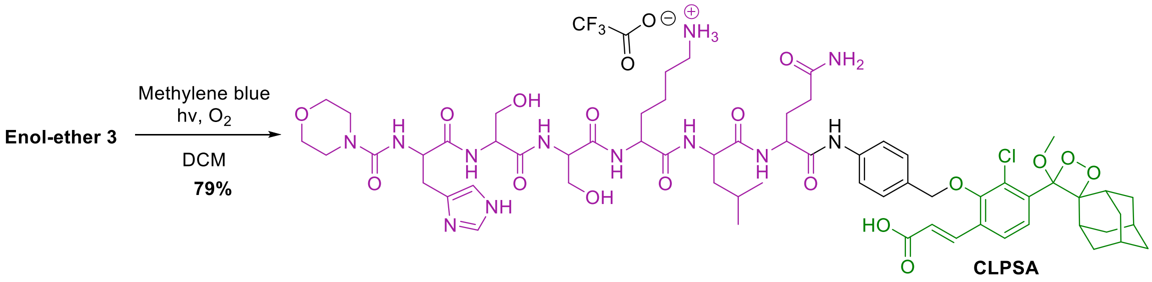

CLPSA

Compound **CLSPA** was synthesized according to a previously reported procedure[2].

MS (ES+): m/z calculated for $[C_{62}H_{86}ClN_{12}O_{17}]^+$: 1304.6; found: 1304.2 $[M]^+$.

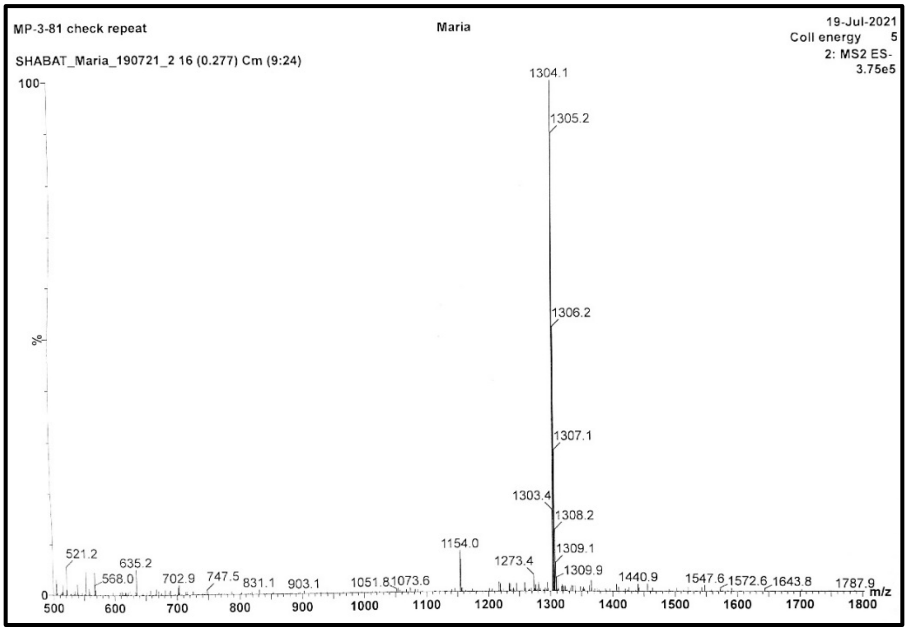

Figure S19. MS(+) spectra of **CLPSA**.

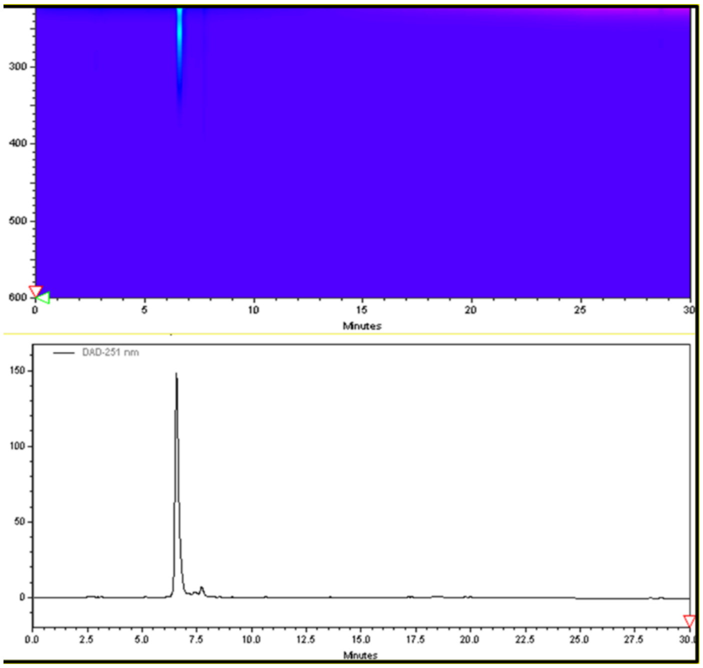

Figure S20. HPLC 2D and 3D chromatogram of **CLPSA** (50-100% ACN:H_2O, RT=5.3 minutes).

NMR spectra

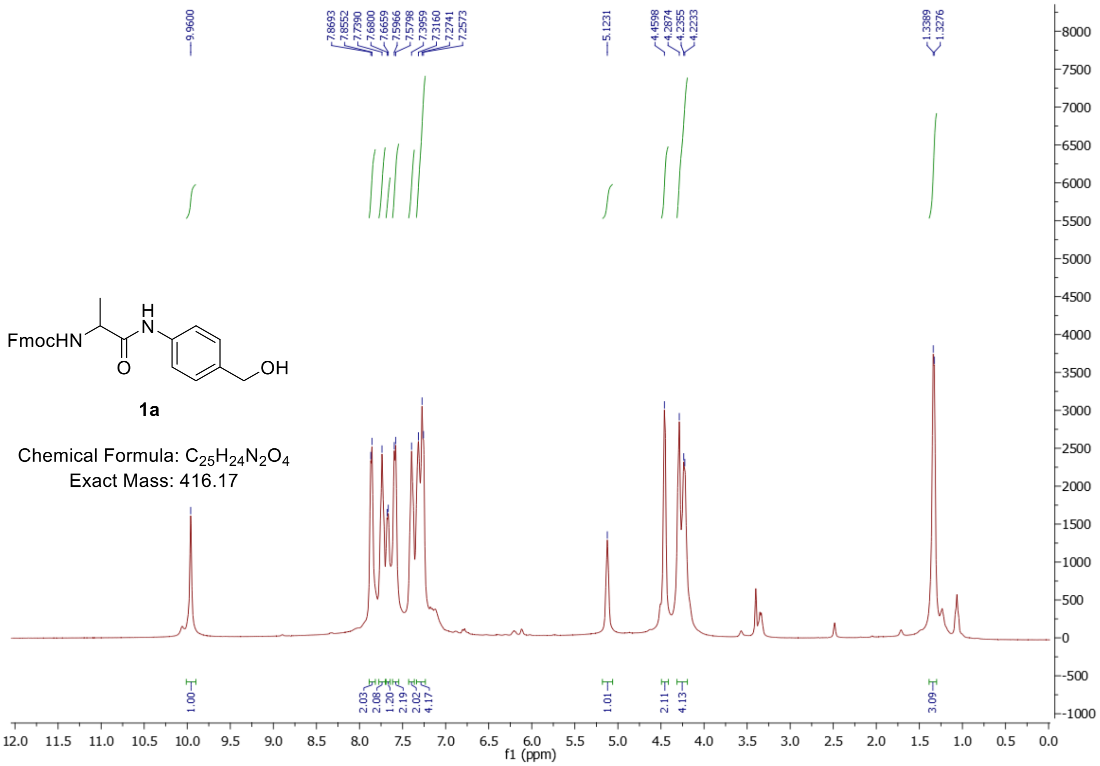

^{1}HNMR spectrum of compound **1a**

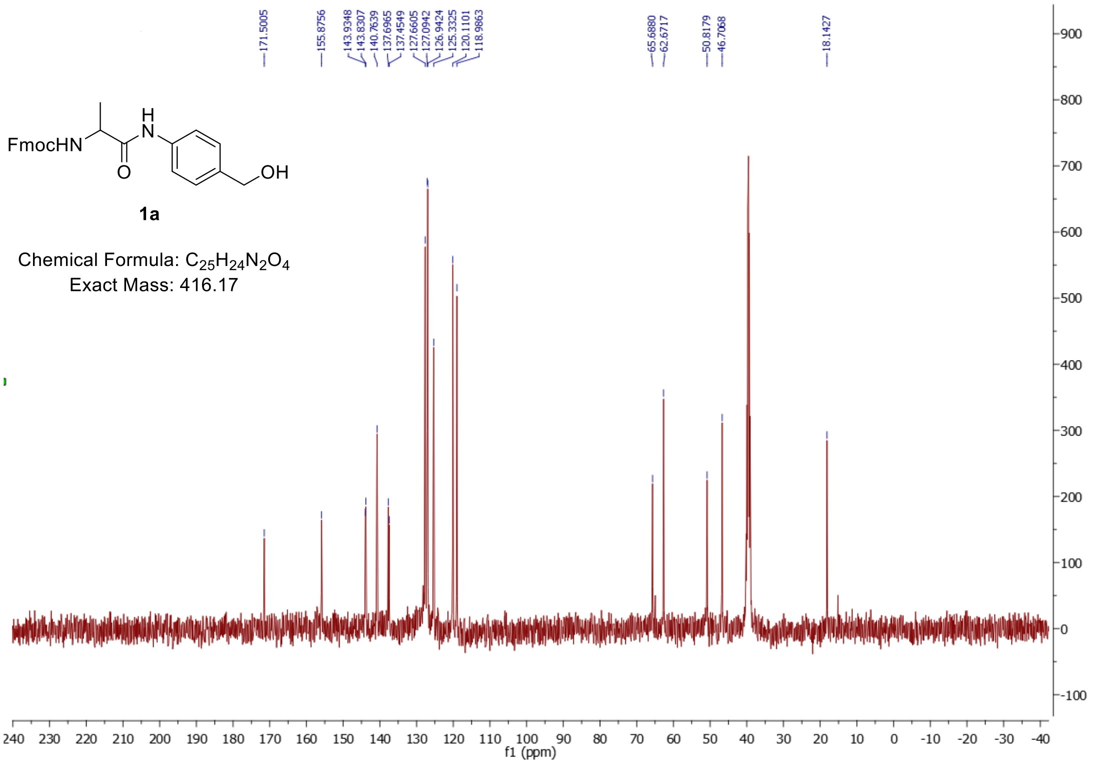

^{13}CNMR spectrum of compound **1a**

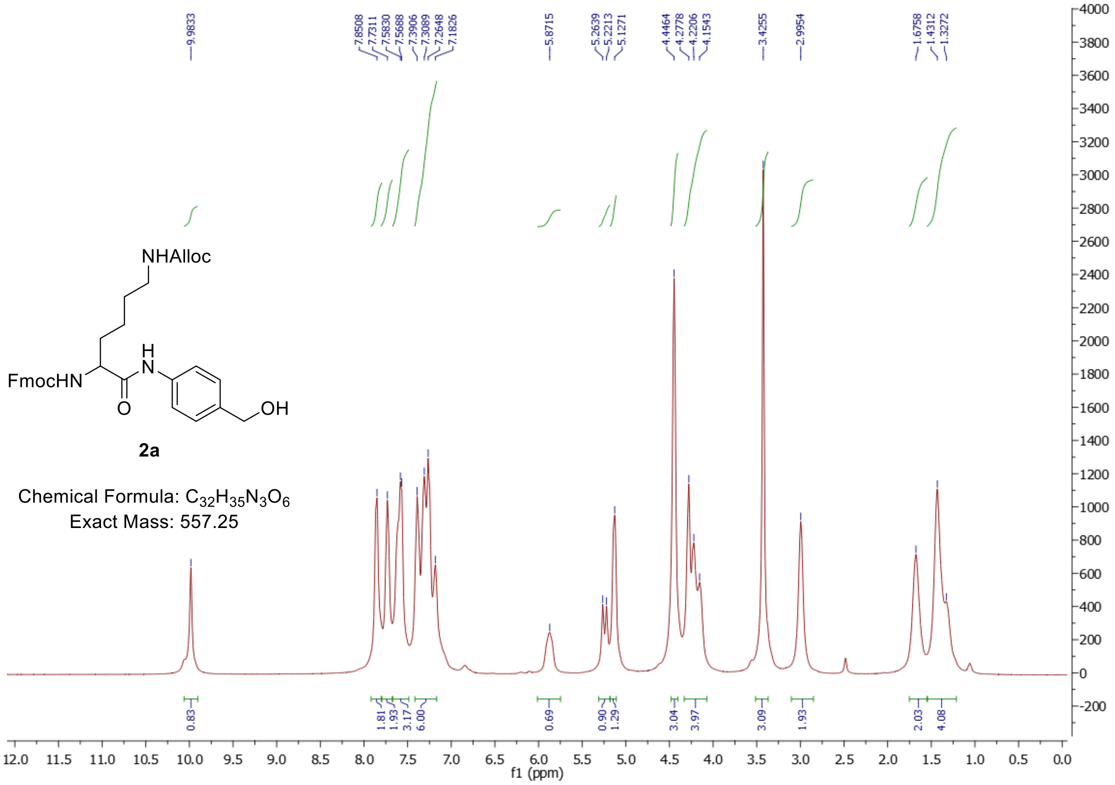

^{1}HNMR spectrum of compound **2a**

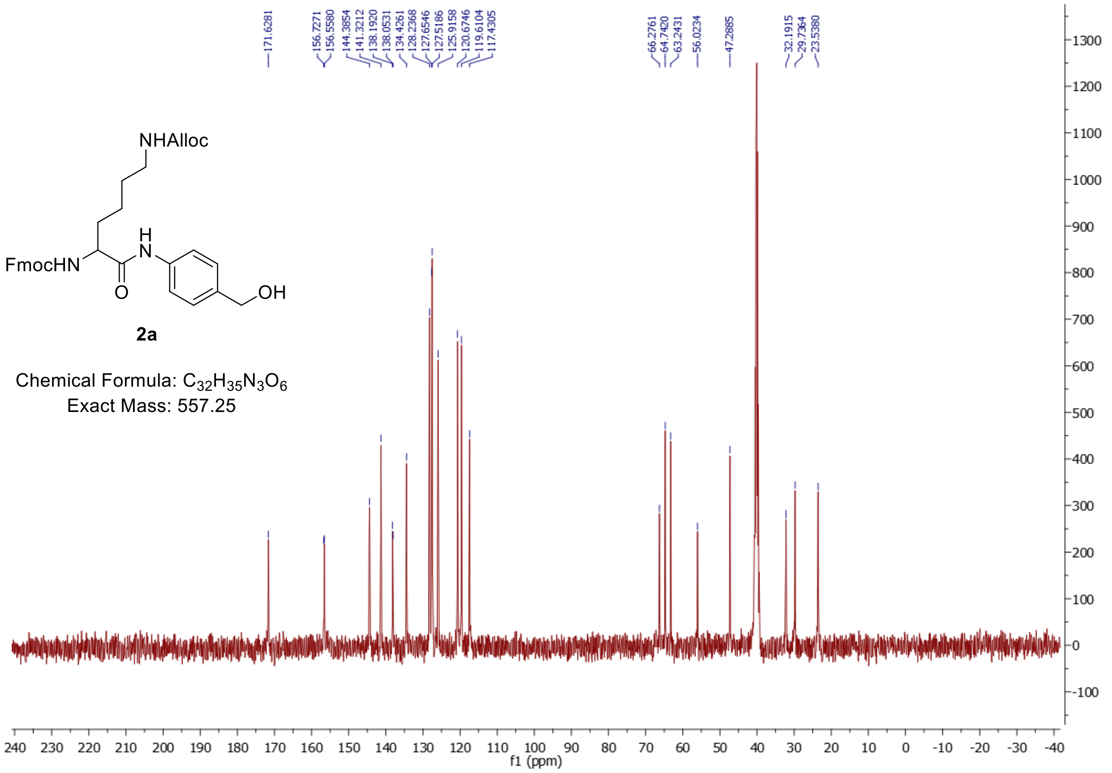

^{13}CNMR spectrum of compound **2a**

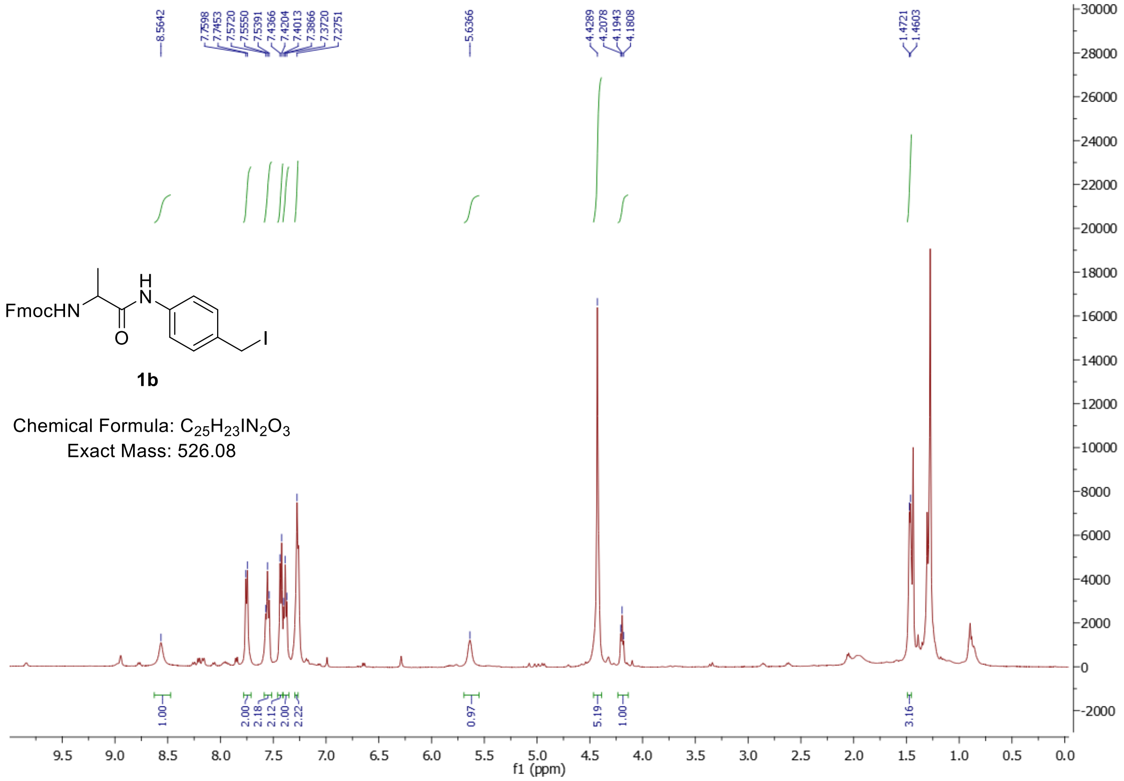

^{1}HNMR spectrum of compound **1b**

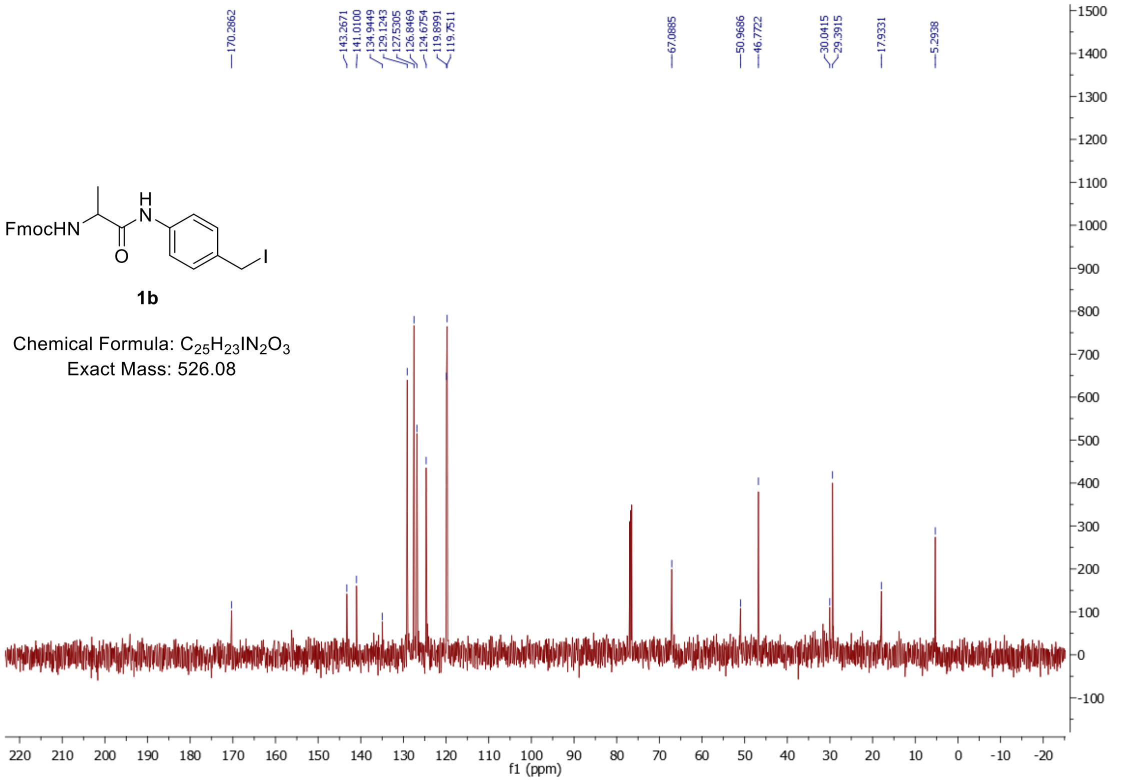

^{13}CNMR spectrum of compound **1b**

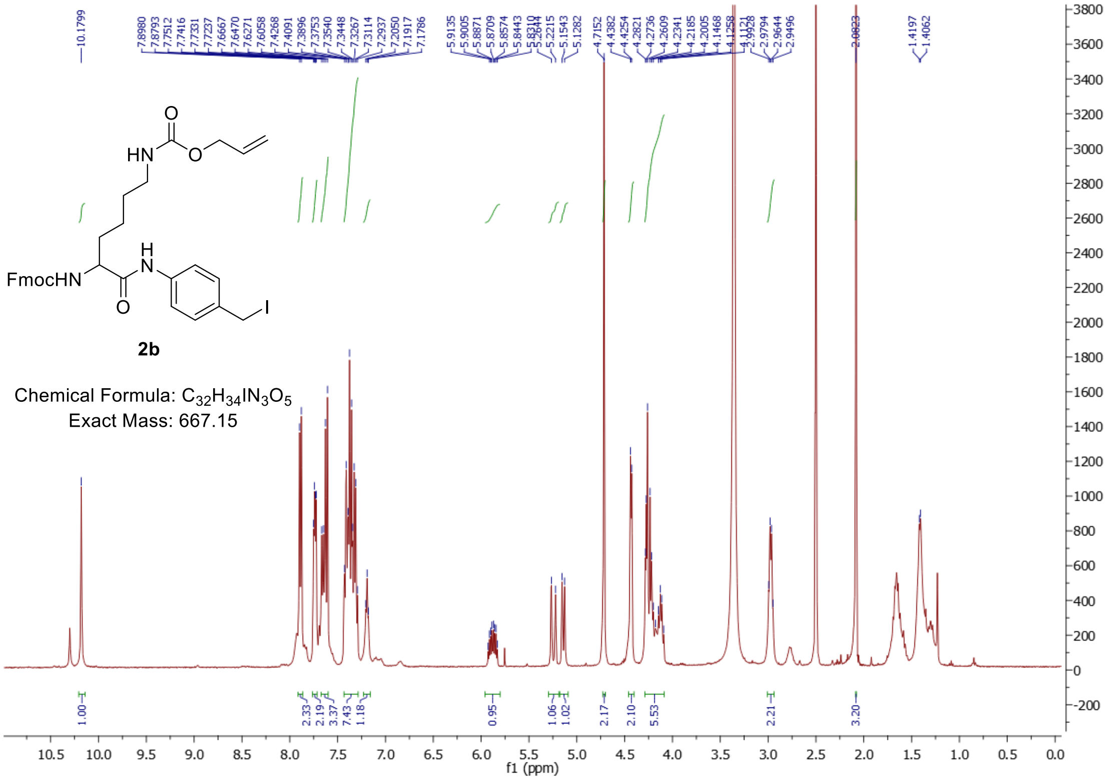

^{1}HNMR spectrum of compound **2b**

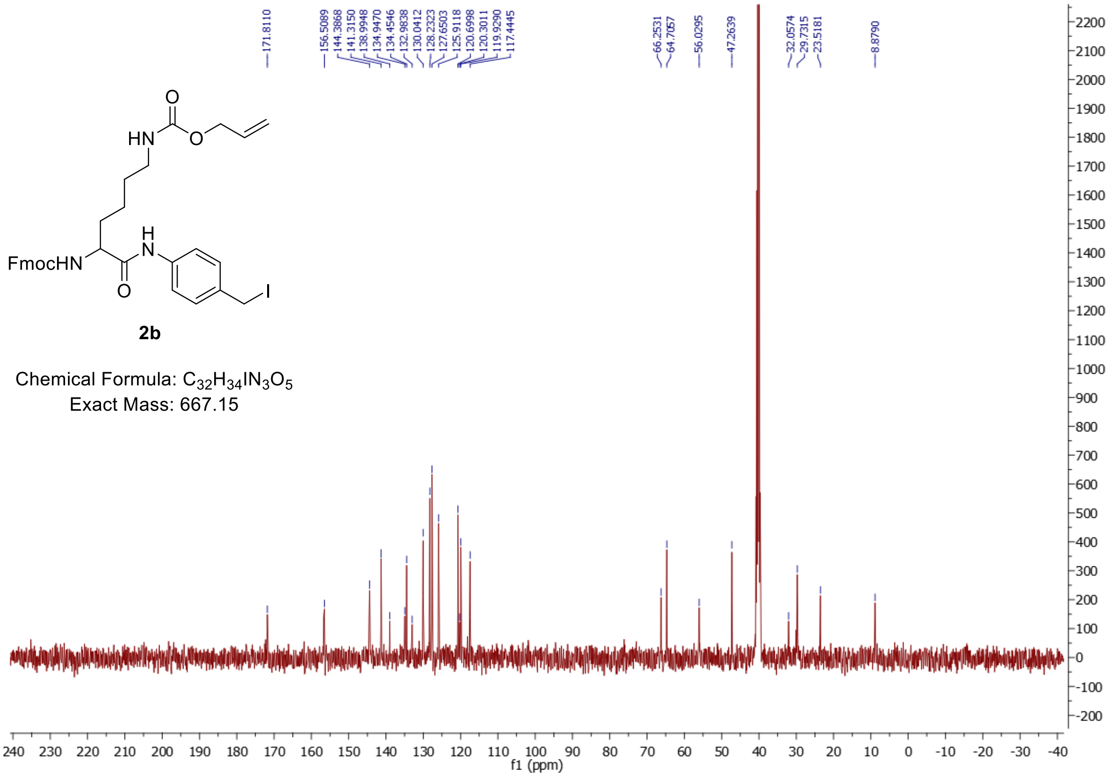

^{13}CNMR spectrum of compound **2b**

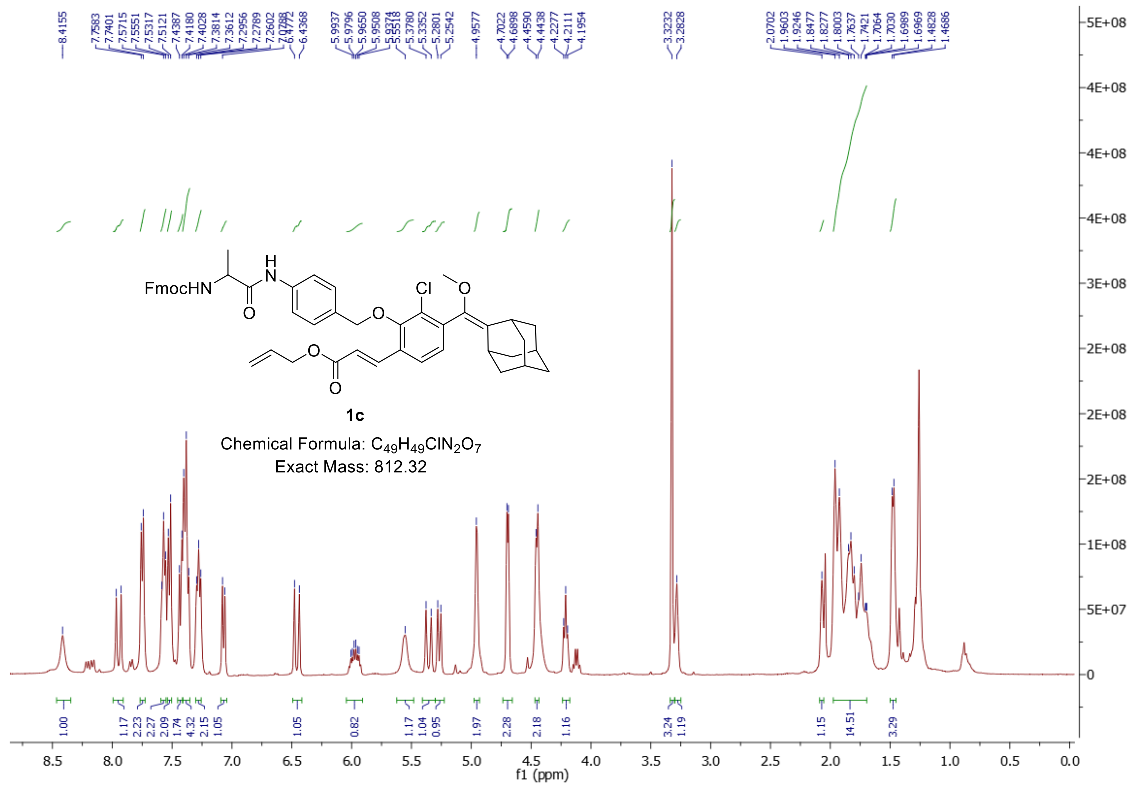

^{1}HNMR spectrum of compound **1c**

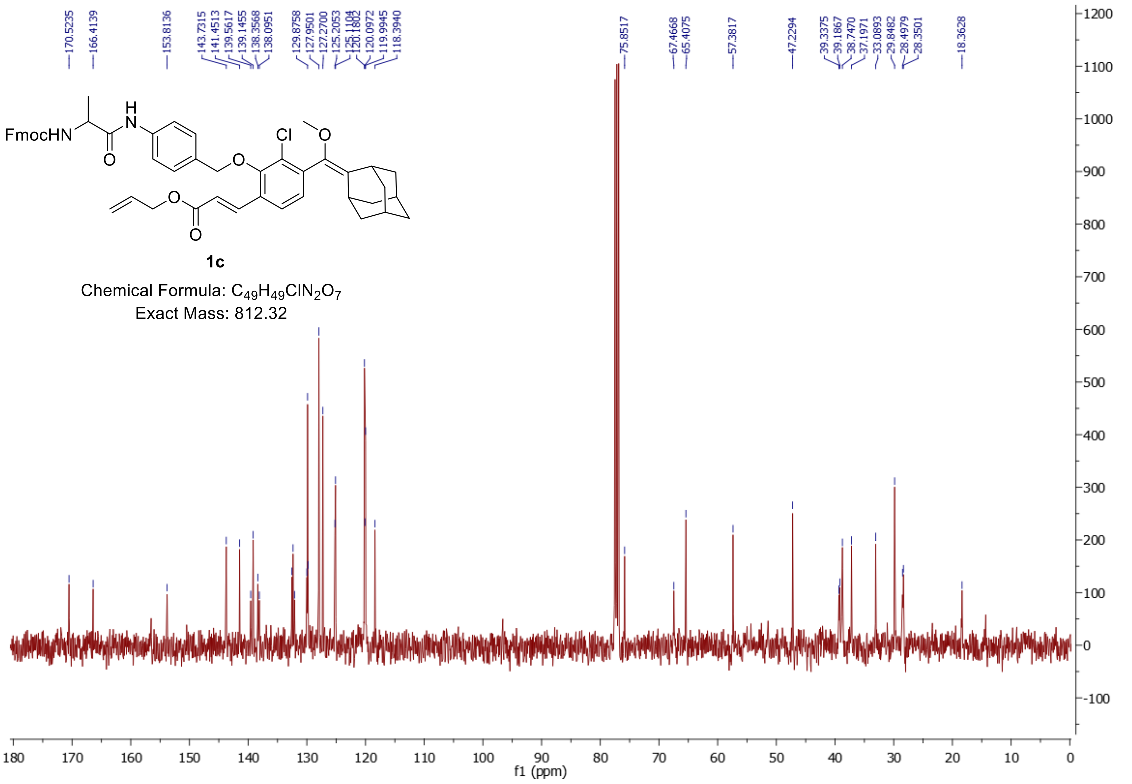

^{13}CNMR spectrum of compound **1c**

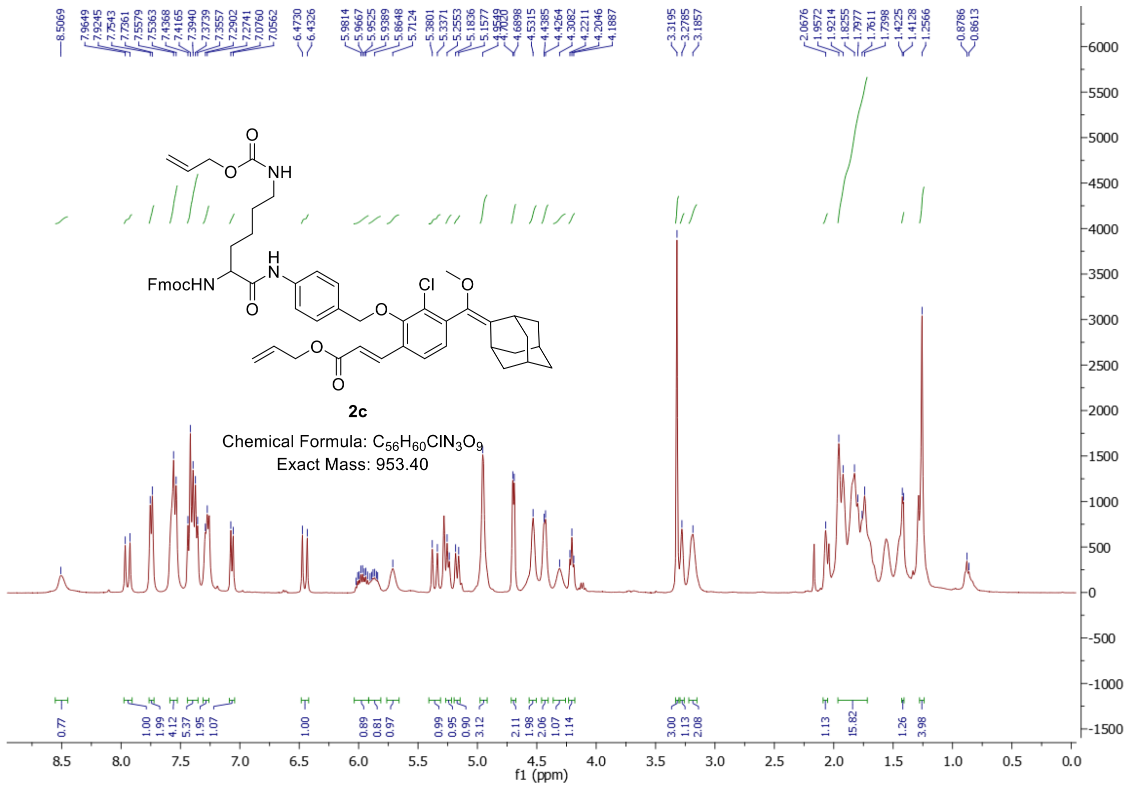

^{1}HNMR spectrum of compound **2c**

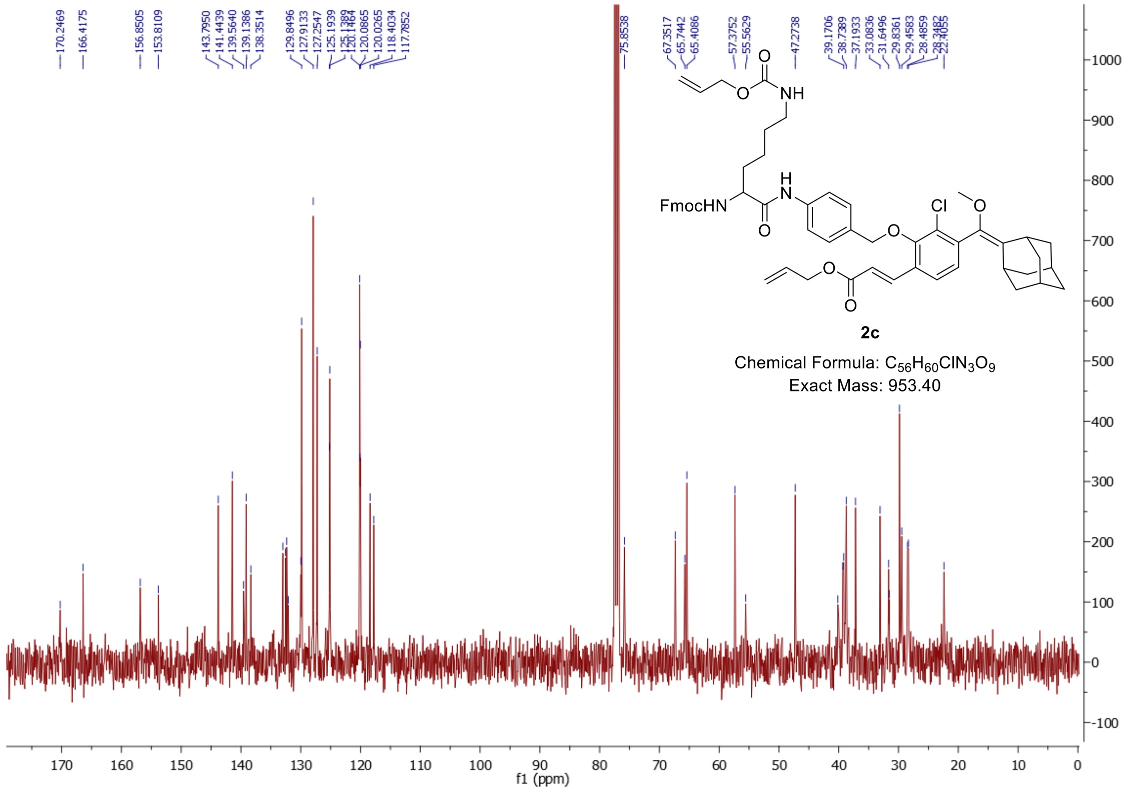

^{13}CNMR spectrum of compound **2c**

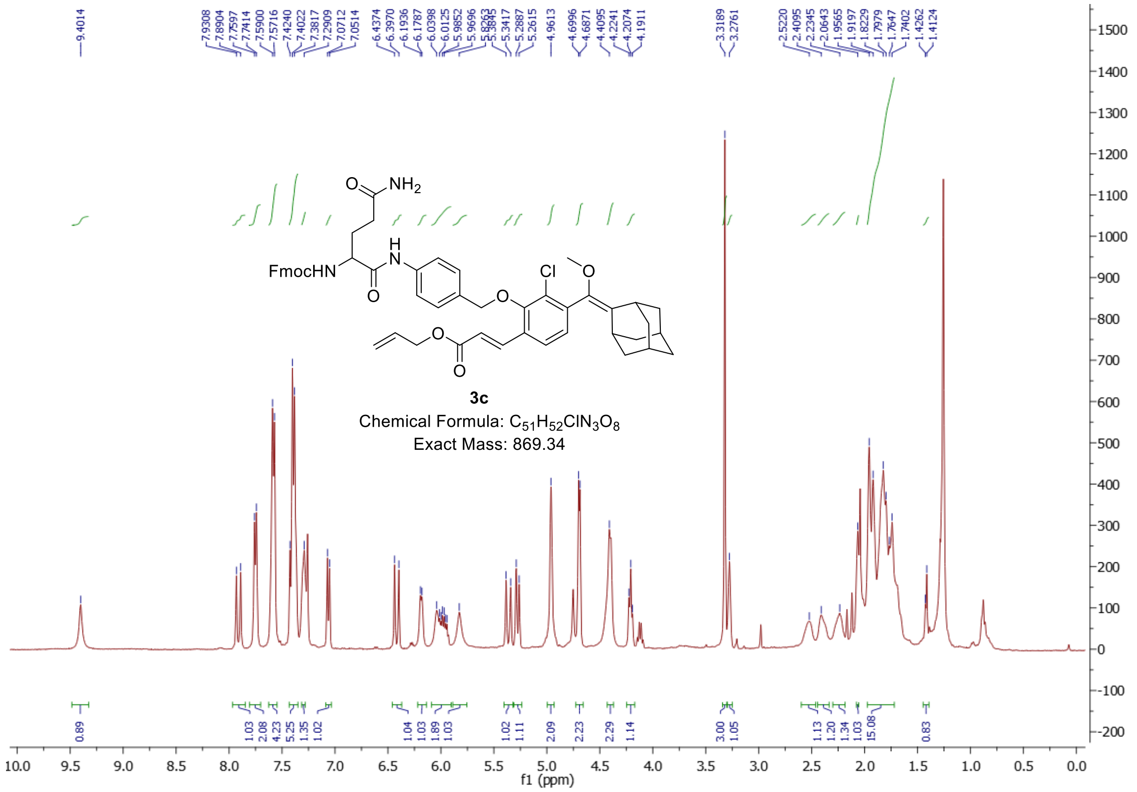

^{1}HNMR spectrum of compound **3c**

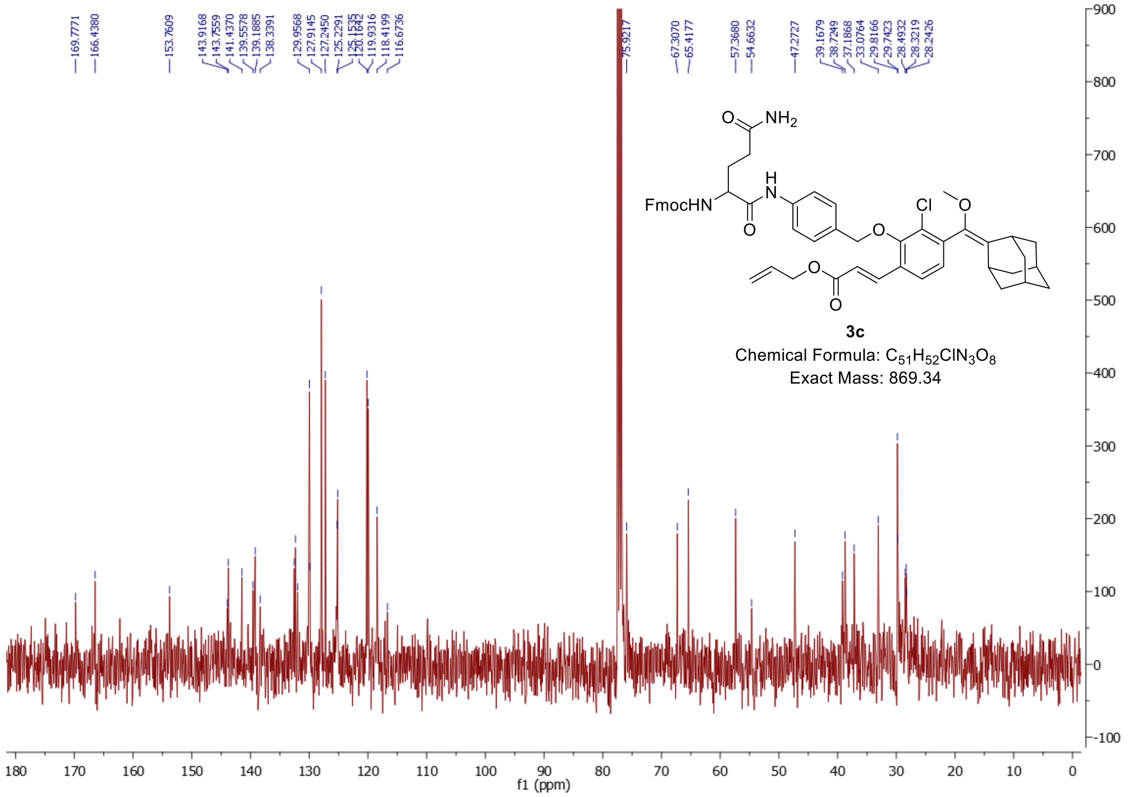

^{13}CNMR spectrum of compound **3c**

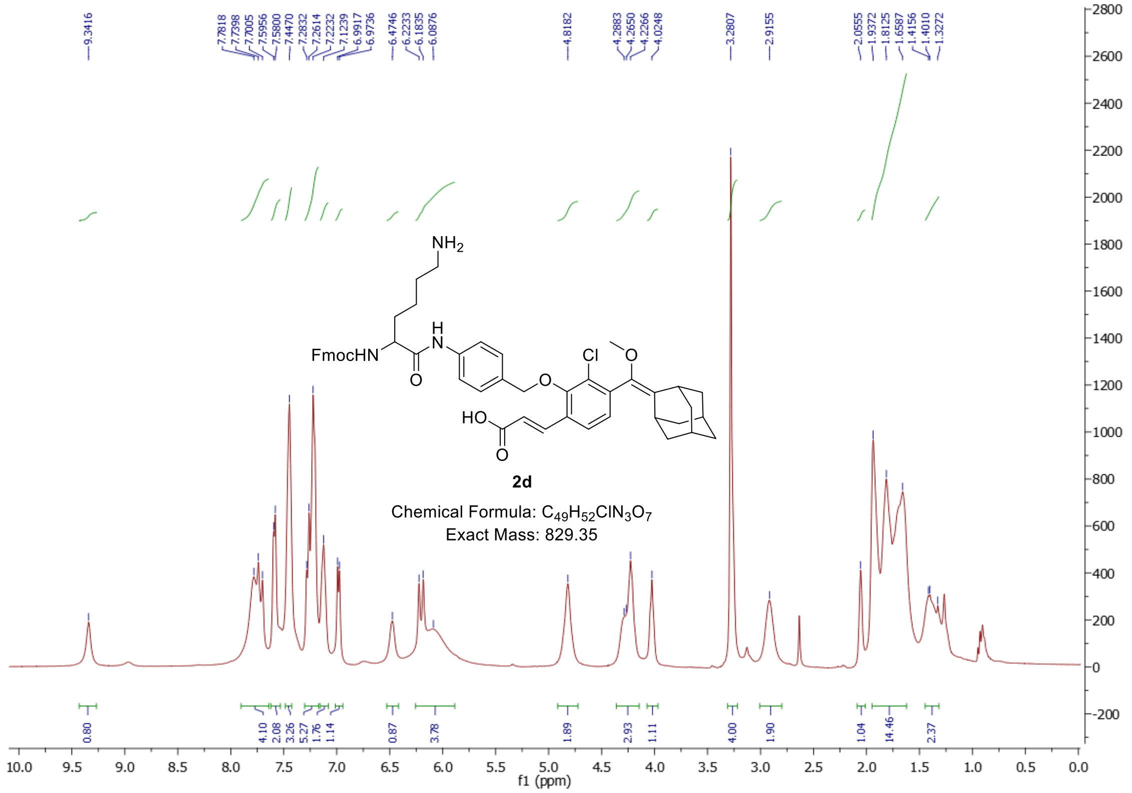

^{1}HNMR spectrum of compound **2d**

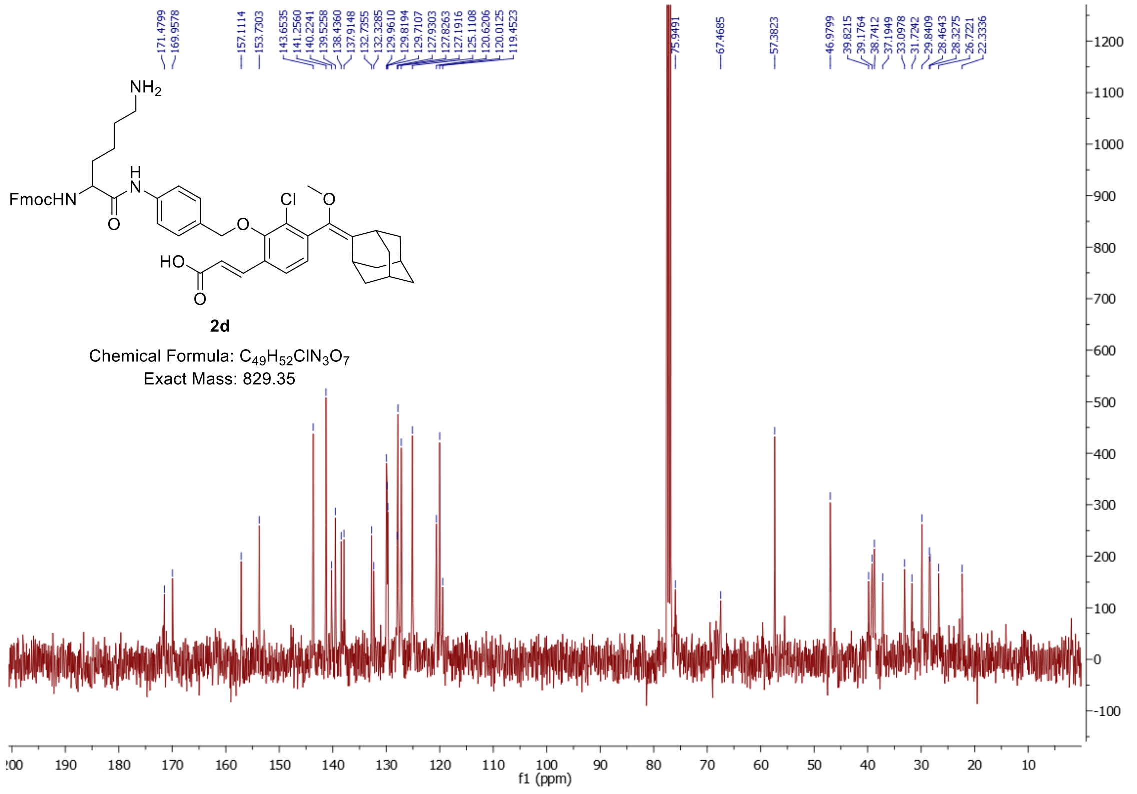

^{13}CNMR spectrum of compound **2d**

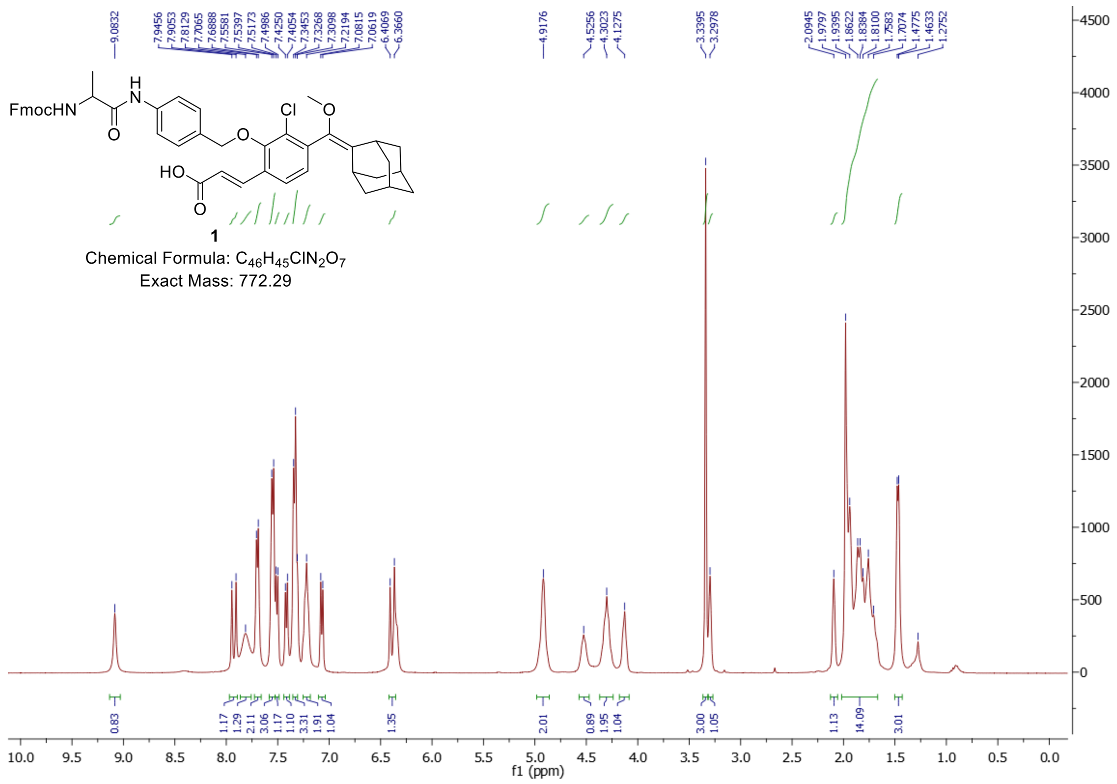

^{1}HNMR spectrum of compound **1**

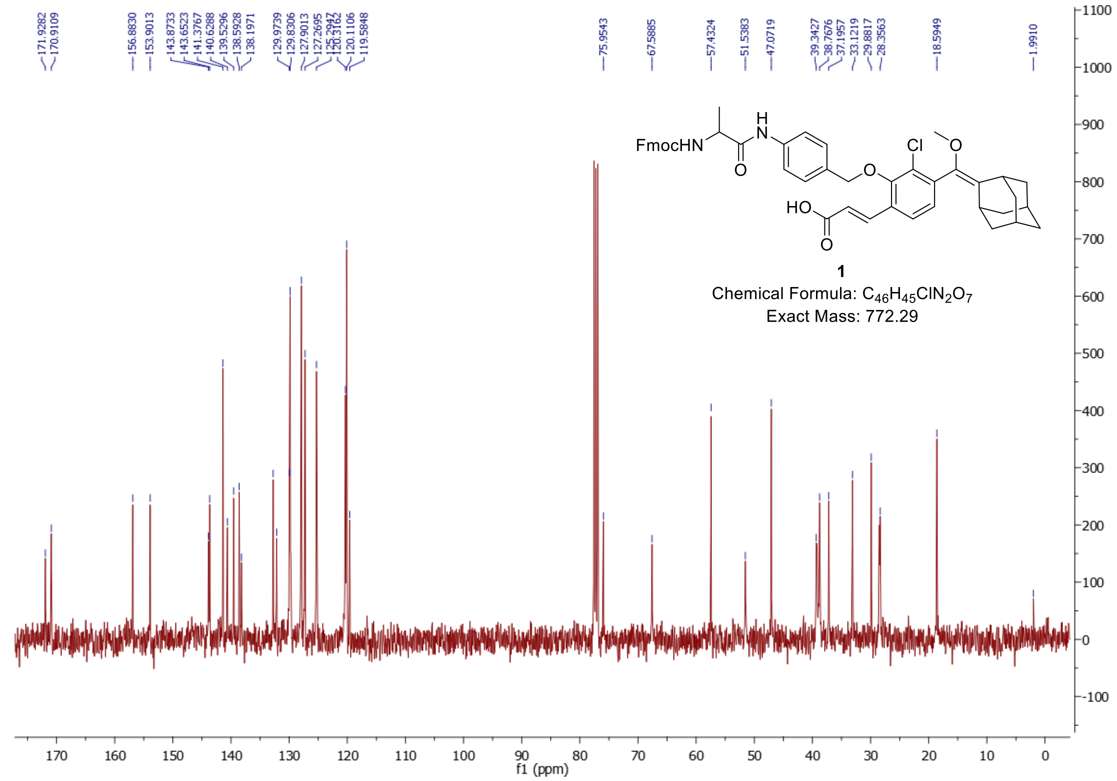

^{13}CNMR spectrum of compound **1**

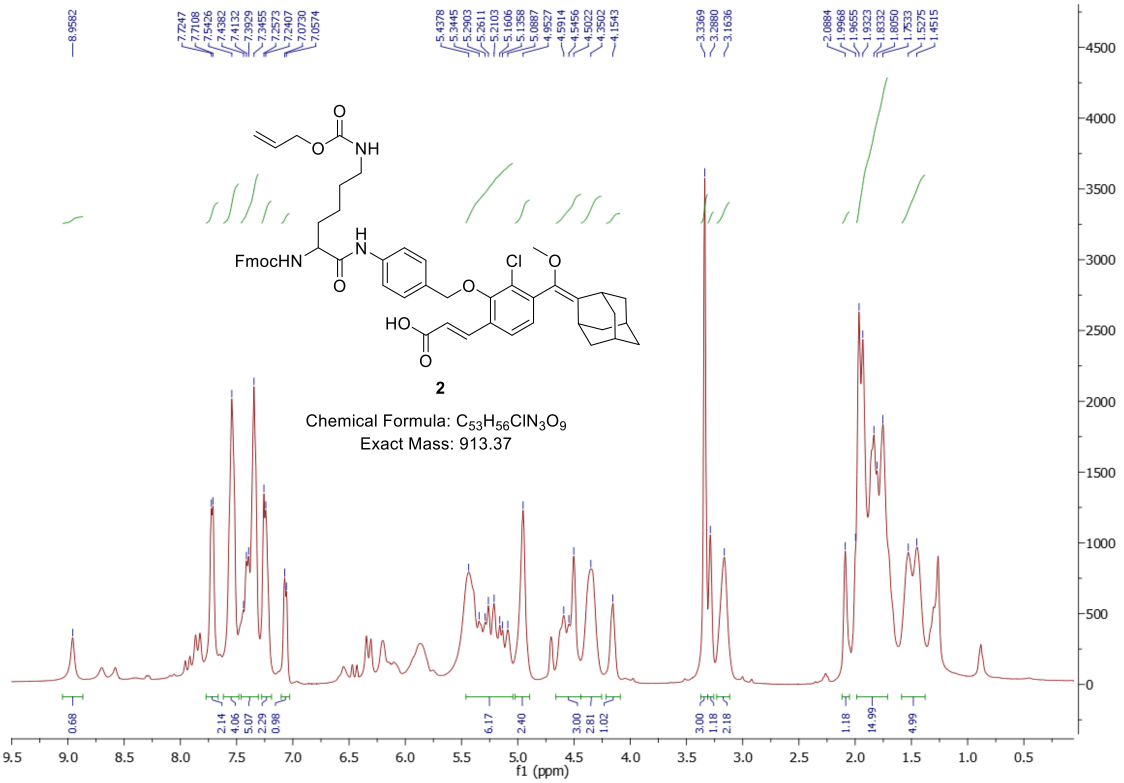

^{1}HNMR spectrum of compound **2**

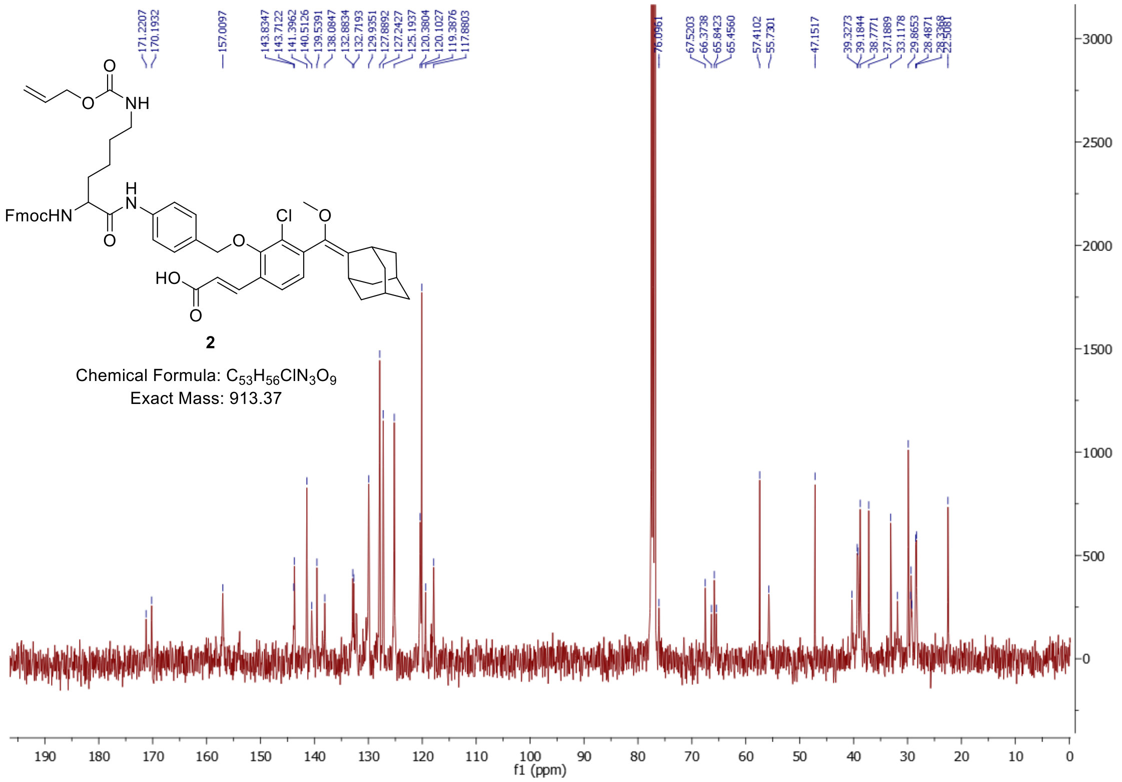

^{13}CNMR spectrum of compound **2**

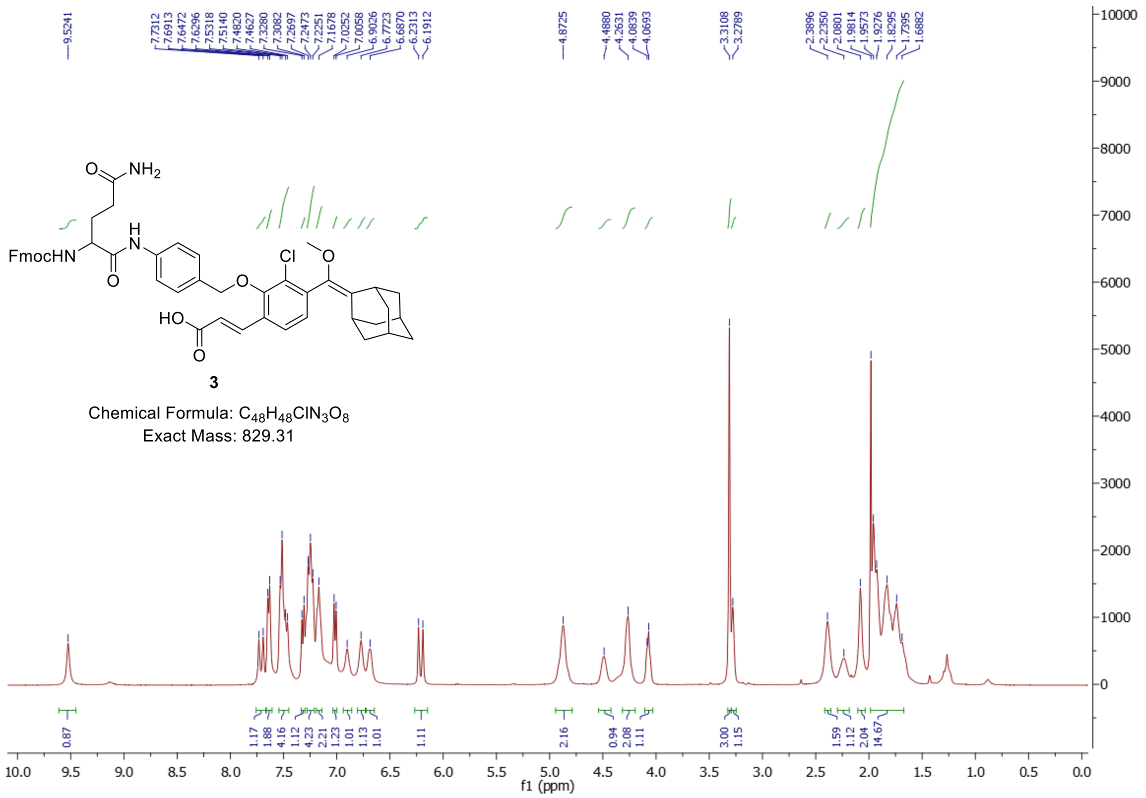

^{1}HNMR spectrum of compound **3**

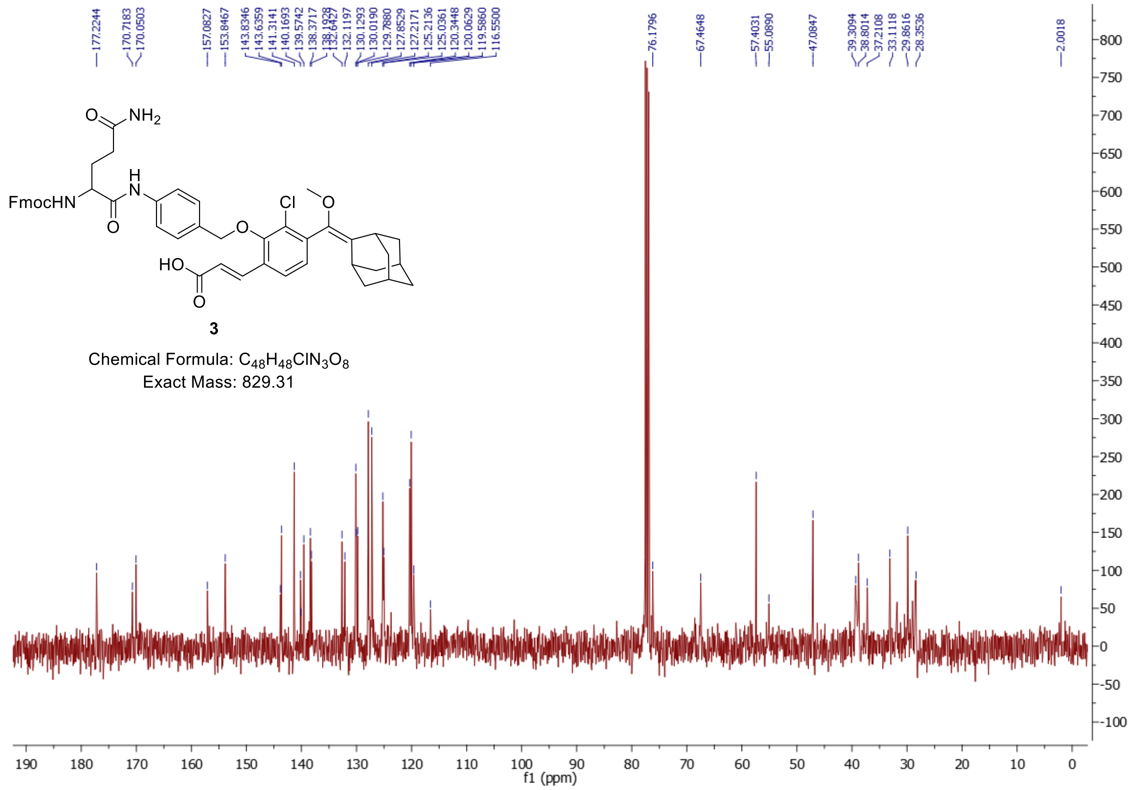

^{13}CNMR spectrum of compound **3**

References

1. Hananya, N., Reid, J.P., Green, O., Sigman, M.S., and Shabat, D. (2019) *Chem. Sci.* 10, 1380-1385.
2. Gutkin, S., Green, O., Raviv, G., Shabat, D., and Portnoy, O. (2020) *Bioconjugate. Chem.* 31, 2488-2493.
3. Roth-Konforti, M., Bauer, C., and Shabat, D. (2017) *Angew. Chem. Int. Ed*. 129, 15839-15844.
4. Kisin-Finfer., Ferber, S., Blau, R., Satchi-Fainaro, R., and Shabat, D. (2014) *Bioorg. Med. Chem. Lett.* 24, 2453-2458.
